# Targeting mechanosensitive cannabinoid receptor 1 with isoflavone prodrugs attenuates atherosclerotic endothelial dysfunction

**DOI:** 10.1186/s12929-026-01214-5

**Published:** 2026-01-21

**Authors:** Dai-Jung Chung, Shao-Peng Chen, Wei-Hsuan Liu, Chia-Yu Liu, Nan-Wei Su, Chen Hsu, Hsin-Ya Tsai, Kai-Chien Yang, Cho-Kai Wu, Sheng-Wei Lin, Jiun‑Jie Shie, Ming-Tao Zhao, Tzu-Tang Wei

**Affiliations:** 1 Department and Graduate Institute of Pharmacology, College of Medicine, National Taiwan University, No. 1, Jen-Ai Road, 1st Section, Taipei, 10051 Taiwan; 2 Department of Agricultural Chemistry, National Taiwan University, Taipei, 106319 Taiwan; 3 Department of Biochemical Science and Technology, National Taiwan University, Taipei, 106319 Taiwan; 4 Division of Cardiology, Department of Internal Medicine, National Taiwan University Hospital, Taipei, 100225 Taiwan; 5 Institute of Biological Chemistry, Academia Sinica, Taipei, 11529 Taiwan; 6 Institute of Chemistry, Academia Sinica, 128 Academia Road, Section 2, Taipei, 11529 Taiwan; 7 Center for Cardiovascular Research, The Abigail Wexner Research Institute, Nationwide Children’s Hospital, Columbus, OH 43210 USA; 8 The Heart Center, Nationwide Children’s Hospital, Columbus, OH 43210 USA; 9 Department of Pediatrics, College of Medicine, The Ohio State University, Columbus, OH 43210 USA; 10 Chemical Biology and Molecular Biophysics, Taiwan International Graduate Program in Chemical Biology and Molecular Biophysics (TIGP-CBMB), Academia Sinica, Taipei, 11529 Taiwan

**Keywords:** Atherosclerotic endothelial dysfunction, cannabinoid receptor 1 (CB1) antagonists, Isoflavone prodrugs, Disturbed flow, Endothelial-to-mesenchymal transition

## Abstract

**Background:**

Despite therapeutic advances, atherosclerosis remains a major global health challenge. Most current treatments target systemic risk factors rather than the diseased vascular wall. Our previous work identified genistein, a soy isoflavone, as a cannabinoid receptor 1 (CB1) antagonist capable of suppressing CB1-mediated vascular inflammation and atherosclerosis. However, its poor water solubility and low oral bioavailability limit clinical application.

**Purpose:**

We aimed to develop water-soluble, orally bioavailable CB1 antagonists for atherosclerosis and to investigate the role of endothelial CB1 in hemodynamic regulation.

**Methods:**

RNA-sequencing datasets from the NCBI GEO repository were analyzed to assess CB1 expression in atherosclerotic patients. Apolipoprotein E-deficient (Apoe^−/−^) mice with or without partial carotid artery ligation (PCAL) were used to model acute and chronic atherosclerosis. A cone-and-plate viscometer was employed to simulate disturbed flow. A ligand-based high-throughput virtual screening approach combined with SWEETLEAD chemical database analysis was used to discover new CB1 antagonists. A biotransformation-based strategy was used to generate isoflavone monophosphate prodrugs.

**Results:**

We found CB1 was upregulated in atherosclerotic lesions from patients and mice, and in endothelial cells exposed to disturbed flow. Mechanistically, this was driven by ZNF610 and Spi1 binding and KLF4 dissociation at the CB1 promoter. Daidzein, a soy isoflavone structurally similar to genistein, was identified as a novel CB1 antagonist. To enhance solubility and bioavailability, we developed genistein 7-O-phosphate (G7P) and daidzein 7-O-phosphate (D7P). Pharmacological treatment with these isoflavone monophosphates or genetic CB1 ablation reversed disturbed flow-induced endothelial dysfunction and endothelial-to-mesenchymal transition (EndMT). Oral administration of G7P and D7P significantly reduced atherosclerotic plaque formation in mice.

**Conclusions:**

This is the first study to identify transcriptional regulators that drive endothelial CB1 upregulation in response to disturbed flow. We further demonstrated that isoflavone monophosphates ameliorate disturbed flow-induced endothelial dysfunction and EndMT via CB1 inhibition, offering promising oral therapeutics for atherosclerosis.

**Supplementary Information:**

The online version contains supplementary material available at 10.1186/s12929-026-01214-5.

## Background

Atherosclerosis is a leading cause of morbidity and mortality worldwide and the primary underlying factor in cardiovascular disease [1]. This chronic inflammatory condition is characterized by the accumulation of lipid-rich plaques in arterial walls, which can lead to serious complications such as myocardial infarction and stroke [2]. Despite therapeutic advancements, current treatments address mainly systemic risk factors such as hypercholesterolemia and hypertension rather than directly targeting the vascular wall [3]. This issue highlights the need to elucidate the endothelial mechanosensitive mechanisms that drive atherosclerosis and to develop targeted therapies for the vascular wall.

Hemodynamic forces play crucial roles in the development of atherosclerosis [4, 5]. Vascular endothelial cells form the inner lining of the blood vessel wall, are directly exposed to blood flow, and perform important homeostatic functions in response to various chemical and mechanical stimuli [6]. Atherosclerotic plaques preferentially form at arterial regions exposed to disturbed flow (DF), such as areas of curvature, branching, and bifurcation, where oscillatory shear stress promotes endothelial dysfunction, inflammation, oxidative stress, mitochondrial dysfunction, and increased vascular permeability [7]. DF-induced endothelial activation contributes to various vascular diseases, including aneurysms, arteriovenous malformations, and atherosclerosis [8]. In contrast, unidirectional laminar flow (LF) in straight arteries results in stable shear stress, which maintains an anti-inflammatory endothelial phenotype and protects against atherosclerosis [9]. The mechanotransduction pathways activated by DF involve mechanosensitive transcription factors such as krüppel-like factor 4 (KLF4), nuclear factor erythroid 2-related factor 2 (NRF2), yes-associated protein (YAP), transcriptional coactivator with PDZ-binding motif (TAZ), TEA domain transcription factors (TEAD), nuclear factor kappa-light-chain-enhancer of activated B cells (NF-κB), and activator protein 1 (AP-1), which differentially regulate vascular pathophysiology [10–12]. Understanding the roles of these mechanosensitive transcription factors in DF-induced endothelial dysfunction may pave the way for innovative strategies to prevent and treat atherosclerosis.

Cannabinoid receptors play crucial roles in metabolic regulation, memory, mood, pain, and immune function [13]. Two cannabinoid receptors, cannabinoid receptor 1 (CB1/CNR1) and cannabinoid receptor 2 (CB2/CNR2), belong to the G protein-coupled receptor (GPCR) superfamily and were identified over 25 years ago [14]. CB1 and CB2 mediate the effects of delta-9-tetrahydrocannabinol (Δ^9^-THC), the main psychoactive component of marijuana, and the subsequently identified endogenous cannabinoids (endocannabinoids) anandamide and 2-arachidonoyl glycerol [15]. CB1, the most abundant GPCR in the mammalian brain, regulates the psychoactive effects of marijuana but is also expressed in peripheral tissues such as the heart, vasculature, and smooth muscle [16]. CB2 is expressed primarily in immune cells, particularly macrophages, but is also expressed in endothelial cells [17]. Unlike classical neurotransmitters, endocannabinoids are made on demand and cause vasodilation, bradycardia, and hypotension [18]. Previous studies have suggested that CB1 activation promotes atherosclerosis by inducing inflammation and oxidative stress, leading to endothelial dysfunction, whereas CB2 activation exerts antiatherogenic effects [19]. As a result, CB2 agonists are in development for vascular disorders, and CB1 antagonists are antiatherogenic [20].

Natural compounds such as polyphenols and isoflavones exhibit a wide range of cardiometabolic and anti-inflammatory activities; however, their therapeutic translation has been greatly limited by poor aqueous solubility and low oral bioavailability [21, 22]. These limitations often lead to insufficient plasma concentrations, requiring chemical modification or prodrug strategies to enhance absorption and stability [23]. To overcome these challenges, ligand-based and structure-guided drug design have emerged as powerful strategies to optimize natural product scaffolds and generate derivatives with improved solubility, potency and overall drug-like properties [24]. Early identification of active compounds is a key step in drug development [25]. The integration of virtual screening with chemical database mining is now widely used in pharmaceutical discovery pipelines [26]. Structure-based virtual screening uses the crystal structure of a target to design lead compounds through large-scale molecular docking experiments, predicting docking poses and binding affinities [27]. Ligand-based virtual screening is based on shape similarity and volume similarity between two compounds [28]. The integration of ligand-based and structure-based approaches is considered more effective than the use of independent models for new drug discovery [29, 30].

Thus, this study was designed to investigate how CB1 is regulated in vascular endothelial cells under disturbed flow and to determine its contribution to endothelial dysfunction and atherosclerosis. We aimed to elucidate the transcriptional mechanisms responsible for disturbed flow-induced CB1 upregulation, with a focus on key flow-responsive transcription factors, including zinc finger protein 610 (ZNF610), transcription factor PU.1 (Spi1), and KLF4. In addition, we sought to identify and develop water-soluble, orally bioavailable CB1 antagonists based on natural isoflavone scaffolds using a combined ligand-based and structure-based virtual screening strategy. The overall goal of this work was to establish endothelial CB1 as a mechanosensitive regulator of atherosclerosis and to develop isoflavone-derived CB1 antagonists as potential oral therapeutics for disturbed flow-induced endothelial dysfunction.

## Methods

### Bioinformatic analysis of GEO datasets

Gene expression data were obtained from the Gene Expression Omnibus (GEO) database (https://www.ncbi.nlm.nih.gov/geo/) under accession numbers GSE21545, GSE40231, and GSE46401. Raw expression matrices and sample annotations were downloaded and processed in R. Data were log-transformed and normalized using standard GEO2R workflows. For datasets containing multiple probes for the same gene, the probe with the highest mean expression was selected. Differential expression between atherosclerotic and non-atherosclerotic tissues was assessed using the limma package. CNR1 expression values were extracted and compared across groups.

### Molecular docking analysis

Protein–ligand docking studies were performed with the crystal structure of human CB1 (PDB: 5TGZ) by Mcule 1-click docking software (Mcule, Palo Alto, CA, USA), and the docking scores of the test compounds were estimated. The Glide module was used to dock the flexible ligands to the rigid binding site without any constraints using the default settings. The resulting GlideScores were used to estimate and rank the binding energy of the ligands. Binding interactions between the test compounds and amino acid residues in the CB1 crystal structure were visualized with Discovery Studio software (version 2024, BIOVIA, San Diego, CA, USA).

### Surface plasmon resonance (SPR) analysis

The binding affinity of daidzein to CB1 protein was assessed using a Biacore T200 surface plasmon resonance instrument (Cytiva, Marlborough, MA, USA). CB1 protein was prepared in sodium acetate buffer (pH 4.5) and immobilized on a CM7 sensor chip with an amine coupling kit (Cytiva, BR100050, Marlborough, MA, USA). Serial two-fold dilutions of daidzein, starting at a concentration of 40 μM, were prepared in running buffer (PBS, pH 7.4, 2% DMSO) and injected into the flow channels at a flow rate of 30 μL/min at 25 °C. Signals were recorded in resonance units (RU), and responses were corrected by subtracting the signal from a reference channel lacking immobilized CB1. Sensorgrams were generated and analyzed using BIAevaluation software (GE Healthcare, Chicago, IL, USA).

### Radioligand binding assays

Radioligand binding assays were performed by Eurofins Discovery (St. Charles, MO, USA) using human recombinant Chem-1 cells expressing the indicated receptors. In these assays, [^3^H]SR141716A (a selective CB1 antagonist), [^3^H]WIN-55,212–2 (a synthetic CB1 agonist), and [^3^H]estradiol (an estrogen receptor ligand) were used as radiolabeled probes. Non-specific binding was determined in the presence of excess unlabeled competitors: CP-55,940 (a high-affinity CB1/CB2 agonist), R( +)-WIN-55,212–2, or diethylstilbestrol, respectively. Cells were incubated at 37 °C for 90 min with the radioligands in assay buffer containing 20 mM HEPES (pH 7.0) and 0.5% bovine serum albumin (BSA). Test compounds were serially diluted in the same buffer. After incubation, membranes were filtered, washed, and retained radioactivity was measured. Half-maximal inhibitory concentrations (IC_50_) were determined by nonlinear least-squares regression using MathIQ software (ID Business Solutions Ltd., GU, UK). Inhibition constants (Kᵢ) were calculated using the Cheng-Prusoff equation based on the measured IC_50_ values, radioligand concentrations, and historical ligand dissociation constants.

### Preparation of genistein 7-O-phosphate and daidzein 7-O-phosphate

G7P and D7P were prepared via a biotransformation process using Bacillus subtilis BCRC 80517, as previously described [31]. Briefly, a 500-mL Hinton flask containing 90 mL of nutrient broth (NB) medium and 1 mL of seed culture was incubated at 37 °C and 120 rpm for 24 h. Subsequently, 10 mL of NB medium supplemented with 10 mM K₂HPO₄, 50 mg MgSO₄·7H₂O, and 50 mg of genistein or daidzein was added to initiate bioconversion. After an additional 24 h of incubation, the culture broth was harvested and centrifuged at 10,000 × g to remove bacterial cells. The supernatant was adjusted to pH 1.0 with concentrated HCl and extracted three times with an equal volume of ethyl acetate. The combined organic layers were dried over anhydrous sodium sulfate and concentrated under reduced pressure. The residue was dissolved in a small volume of acidified distilled water (pH 1.0) and loaded onto a prepacked DIAION HP-20 resin column equilibrated with acidified water. After extensive washing to remove impurities, adsorbed compounds were eluted stepwise with aqueous methanol of increasing concentrations. The 10% methanol fraction enriched in G7P or D7P was collected, dissolved in 50% methanol, and further purified using a semipreparative reverse phase high performance liquid chromatography (RP-HPLC) system (Waters 600E, Waters, Milford, MA, USA) with a Hypersil ODS C18 column (250 × 10 mm, 10 µm) and a Waters 486 UV/Vis detector. The mobile phase consisted of 0.2% formic acid in water (solvent A) and acetonitrile containing 0.2% formic acid (solvent B). After injection of a 100-µL sample, solvent B was increased from 20 to 40% over 20 min, then to 80% over 5 min, reduced back to 20% within 5 min, and held for an additional 5 min at a flow rate of 5 mL/min at 30 °C. The purified products were lyophilized and used in subsequent experiments. The purity of G7P and D7P powders was confirmed by analytical RP-HPLC, and structural identification was performed by mass spectrometry.

### RP-HPLC analysis of genistein 7-O-phosphate and daidzein 7-O-phosphate.

The RP-HPLC system used for the analysis of isoflavone substrates (genistein and daidzein), their phosphate derivatives in biotransformation broths, and the purity assessment of the purified powders consisted of a Hitachi L-2130 module (Hitachi Ltd., Tokyo, Japan) equipped with a YMC-Pack ODS-AM C18 column (250 × 4.6 mm, 5 μm) protected by a guard cartridge (Hichrom 5C18, Berkshire, UK), and a Hitachi L-2420 UV detector (Hitachi Ltd., Tokyo, Japan). The mobile phase was a linear gradient of 0.1% phosphoric acid in ddH₂O (solvent A) and acetonitrile (solvent B) at 30 °C, with a flow rate of 1.0 mL/min. Following injection of a 20 μL sample, solvent B was increased from 15 to 25% over 5 min, then to 40% over the next 5 min, and subsequently to 45% over the following 10 min. The gradient was then returned to 15% solvent B within 5 min and held for an additional 5 min, yielding a total run time of 30 min. Eluted compounds were monitored at 254 nm. Chromatographic data were analyzed using the SISC Chromatography Data Station v3.1 (Scientific Information Service, Taipei, Taiwan), and quantification was performed using an external standard calibration method.

### Mice

Mice were obtained from the National Laboratory Animal Center (Taipei, Taiwan). All animal experiments were approved by the Institutional Animal Care and Use Committee (IACUC) of the College of Medicine, National Taiwan University (Approval No. 20220024, approved on February 24, 2022).

### Mouse model of partial carotid artery ligation

Male C57BL/6-ApoE^em1Narl^/Nail (Apoe^−/−^) mice were purchased from the National Laboratory Animal Center (Taiwan). The procedure was performed as previously described [32]. An accelerated carotid atherosclerosis model was established through partial carotid artery ligation (PCAL) in Apoe^−/−^ mice fed a 45% high-fat diet (HFD; 45% kcal from fat, primarily from lard; 4.73 kcal/g; D12451, Research Diets, New Brunswick, NJ, USA). Apoe^−/−^ mice were anesthetized via the intraperitoneal injection of 250 mg/kg 2,2,2-tribromoethanol. After shaving, a ventral midline incision (4 to 5 mm) was made in the neck. The left carotid artery was exposed through blunt dissection. Three of the four caudal branches of the left carotid artery (LCA), including the left external carotid artery, were ligated to the left carotid, left internal carotid, and occipital arteries, while the superior thyroid artery remained intact. One day after PCAL, the mice were divided into four groups: (1) vehicle control (n = 5), (2) G7P (5 mg/kg; n = 5), (3) D7P (5 mg/kg; n = 5), and (4) G7P + D7P (n = 5). All the groups received Δ^9^-THC (1 mg/kg) via intraperitoneal injection twice daily for 10 days. Throughout the 12-day treatment period, the mice were maintained on a HFD. After sacrifice, the LCA and right carotid artery (RCA) were collected for use in the subsequent experiments.

### Mouse model of osmotic minipump implantation

Male Apoe^−/−^ mice were fed a 45% HFD (D12451, Research Diets, New Brunswick, NJ, USA) starting at 8 weeks of age for 12 weeks to accelerate atherosclerotic plaque formation. The mice were anesthetized with 2.5% isoflurane mixed with oxygen, and the implant site was shaved before a small subcutaneous incision was made. Osmotic minipumps (ALZET, Model 2006; DURECT Corporation, Cupertino, CA, USA) containing 200 µl of Δ^9^-THC (1 mg/kg/day) were implanted. At 6 weeks, a second pump was implanted. G7P (5 mg/kg), D7P (5 mg/kg), or their combination was administered orally once daily using a feeding needle. At the end of 12 weeks, the mice were euthanized, and saline perfusion was performed. The aorta, heart, and carotid arteries were harvested en bloc. The mice were monitored and weighed daily throughout the experimental period.

### Analysis of plaque lesions in the aorta and carotid arteries

The aorta and carotid arteries were immersed in a 30% sucrose solution for 24 h, followed by incubation in a 1:1 mixture of optimal cutting temperature (OCT) compound and 30% sucrose solution for 1 h. Frozen-embedded samples were sectioned at a thickness of 8 µm using a cryostat and stained with an Oil Red O Staining Kit (Sigma-Aldrich, MAK194, Burlington, MA, USA). The plaque area was quantified using ImageJ software, and the extent of atherosclerosis was expressed as the plaque area on each arterial section.

### Hematoxylin and eosin (H&E) staining

At the time of sacrifice, carotid artery tissues were collected from Apoe^−/−^ mice and processed for histological analysis. Briefly, tissues were fixed in 4% paraformaldehyde (PFA) overnight, transferred to 70% ethanol for 24 h, embedded in paraffin, and sectioned at 5 μm thickness. Sections were stained with hematoxylin for 3–5 min, rinsed in running tap water, and briefly differentiated in acid alcohol. After bluing in ammonia water, sections were counterstained with eosin for 1–2 min, dehydrated through graded ethanol, cleared in xylene, and mounted with coverslips.

### Immunofluorescence staining

En face immunofluorescence staining of the vascular endothelium in the mice was performed as previously described [32]. Briefly, frozen samples were sectioned at 8 µm thickness and permeabilized with 0.1% Triton X-100 for 10 min at room temperature. The cell samples were fixed with 4% PFA for 15 min and blocked with 5% goat serum for 1 h at room temperature. Both the tissue and cell samples were then incubated with specific antibodies at 4 °C for 16 h. After washing, the samples were exposed to Alexa Fluor 488- or Cy3-conjugated secondary antibodies for 1 h at room temperature. Nuclei were stained with DAPI. Fluorescence images were captured using the EVOS M7000 Imaging System (Thermo Fisher Scientific, Waltham, MA, USA) and analyzed with ImageJ software. Detailed antibody information is provided in Table S2.

### Shear stress exposure

An atherorelevant cone-plate flow system was used to generate shear stress in endothelial cells, as described previously [33]. Endothelial cells (HUVECs, HAECs, and hiPSC-ECs) were seeded in six-well plates at a density of 3 × 10^5^ cells per well and cultured in EGM-2 medium supplemented with 4% dextran to achieve 100% confluence. The cells in the center of the well were exposed to disturbed flow conditions characterized by low-magnitude shear stress (3 dynes/cm^2^) with rapid directional variations. In contrast, the cells at the periphery of the well were subjected to laminar flow conditions, characterized by high-magnitude wall shear stress (12 dynes/cm^2^) in a relatively uniform direction. Both flow conditions were maintained for 24 h under standard culture conditions. Detailed information regarding cell line sources and culture media compositions is provided in the Supplementary Information.

### Real‑time polymerase chain reaction (qPCR)

Total RNA was extracted using TRIzol Reagent (Thermo Fisher Scientific, 15596026). RNA concentration and purity were assessed by spectrophotometry (A260/280), and RNA integrity was verified by agarose gel electrophoresis. cDNA was synthesized from 2 μg of total RNA using oligo-dT primers and reverse transcriptase (Thermo Fisher Scientific) following the manufacturer’s protocol. qPCR was performed using SYBR Green Master Mix on an ABI Prism 7900 system (Applied Biosystems, San Francisco, CA, USA) under the following cycling conditions: 95 °C for 10 min, followed by 40 cycles of 95 °C for 15 s and 60 °C for 1 min. Gene expression levels were calculated using the 2^–ΔΔCt^ method and normalized to GAPDH. Primer sequences are shown in Table S1.

### Chromatin immunoprecipitation and quantitative PCR (ChIP‑qPCR) assay

HUVECs were subjected to crosslinking with 2% formaldehyde for 15 min, quenched, collected, and lysed in 1 mL IP buffer (150 mM NaCl, 50 mM Tris–HCl pH 7.5, 5 mM EDTA, 0.5% NP-40, 1% Triton X-100) containing protease inhibitors. The obtained nuclear pellet was resuspended in IP buffer and sonicated. ChIP assays were performed using IgG isotype control and specific antibodies. The immunoprecipitated DNA and input DNA were extracted by incubating the samples with 100 μL of 10% Chelex (Bio-Rad Laboratories, 1421253, Hercules, CA, USA), boiling them to reverse crosslinking and then centrifuging them to remove the Chelex slurry. qPCR was performed using SYBR Green Master Mix with the following thermal cycling conditions: 95 °C for 10 min, then 40 cycles of 95 °C for 15 s and 60 °C for 1 min. ChIP-qPCR primers for the CNR1 promoter were: forward 5’-AGGAACAGAATGCGTGCACCTCTA-3’ and reverse 5’- AACATGAAGCCTCTTTCCCAGCCA-3’.

### Western blot analysis

Protein samples were extracted using lysis buffer containing 100X protease and phosphatase inhibitors and quantified with the Pierce Bradford Protein Assay Kit (Thermo Fisher Scientific, 23200). Protein concentration was measured using an Enzyme-Linked Immunosorbent Assay (ELISA) reader. Cells were lysed on ice, and total cell lysates were centrifuged at 13,000 rpm for 15 min at 4 °C. The lysates were then subjected to SDS-PAGE using appropriately percentage polyacrylamide gels. Detailed antibody information is provided in Table S2.

### Alkaline phosphatase (ALP) activity assay

ALP activity was measured using the Alkaline Phosphatase Fluorometric Assay Kit (Abcam, ab83371, Cambridge, UK), which quantifies the cleavage of nonfluorescent 4-methylumbelliferyl phosphate disodium salt by ALP. Various types of endothelial cells (HAECs, HUVECs, and hiPSC-ECs) were exposed to different shear stress conditions for 24 h. Afterward, 120 μl of conditioned medium from each well was collected, and ALP activity was measured according to the manufacturer’s instructions.

### Cyclic adenosine monophosphate (cAMP) measurement

The cAMP concentration was measured via the cAMP-Glo™ Assay Kit (Promega Corporation, V1501, Madison, WI, USA). Endothelial cells (HUVECs, HAECs, and hiPSC-ECs) were seeded into 6-well plates and exposed to shear stress as previously described. Afterward, the cells were harvested with trypsin and reseeded into 96-well plates, and cAMP levels were measured according to the manufacturer’s instructions. To prevent cAMP degradation during sample preparation, the assay was performed in the presence of the phosphodiesterase inhibitor 1-methyl-3-isobutylxanthine (IBMX), which is included in the kit’s lysis/working buffer.

### Statistical analysis

Data were obtained from at least three independent biological replicates with three technical replicates each. Data normality was assessed using the Shapiro–Wilk test, and homogeneity of variance was evaluated using Levene’s test. For normally distributed data with equal variance, results are presented as mean ± SEM and analyzed using Student’s t-test, one-way ANOVA, or two-way ANOVA with Tukey’s post hoc test where appropriate. For non-normally distributed data, results are presented as median with interquartile range (IQR) and analyzed using the Mann–Whitney U test. Statistical analyses were performed using GraphPad Prism 8. P values < 0.05 were considered statistically significant.

## Results

### Endothelial CB1 expression is elevated in atherosclerotic lesions

**Fig. 1:**
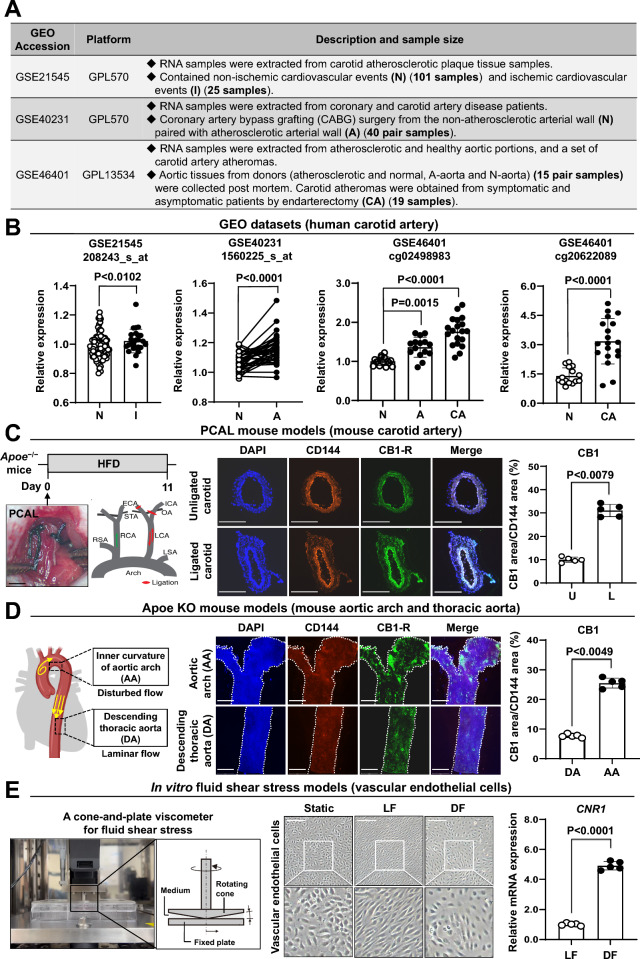
Expression levels of CB1 in atherosclerosis. (A and B) CNR1 expression levels in human arteries. **A** Summary of individual microarray datasets retrieved from the GEO database (https://www.ncbi.nlm.nih.gov/gds). GPL570, (HG-U133_Plus_2) Affymetrix Human Genome U133 Plus 2.0 Array. GPL13534, Illumina HumanMethylation450 BeadChip (HumanMethylation450_15017482). **B** Microarray data from the GSE21545, GSE40231, and GSE46401 datasets, which include patients with carotid atherosclerotic plaques and carotid artery disease, were analyzed. CNR1 expression levels are shown. (C and D) CB1 expression levels in mouse arteries. **C** Schematic overview of the experimental design (upper left panel). Carotid artery sections from partial carotid artery ligation (PCAL) model mice (n = 5) were analyzed, and the unligated (U) and ligated (L) carotid arteries were compared. A diagram of partial ligation of the carotid artery is shown (lower left panel). Scale bar: 5 mm. The left common carotid artery (LCA), external carotid artery (ECA), internal carotid artery (ICA), and superior thyroid artery (STA) were ligated, leaving the occipital artery (OA) open. The right subclavian artery (RSA), right common carotid artery (RCA), and left subclavian artery (LSA) were unligated and remained patent. Immunofluorescence staining was performed using specific antibodies (middle panel). Scale bar: 250 μm. Quantification of the CB1-positive area within carotid artery sections is shown (right panel). **D** Schematic illustration of the aortic arch and descending thoracic aorta (left panel). Arterial sections from Apoe^−/−^ model mice (n = 5) were analyzed, and the aortic arch (AA) and descending thoracic aorta (DA) were compared. En face immunofluorescence staining was performed using specific antibodies (middle panel). Scale bar: 20 μm. The quantification of the CB1-positive area within arterial sections is presented (right panel). **E** CNR1 expression levels in vascular endothelial cells. Schematic illustration of the cone-and-plate viscometer used to generate fluid shear stress in vascular endothelial cells (left panel). Morphological changes in hiPSC-ECs were observed under laminar flow (12 dyne/cm^2^), disturbed flow (3 dyne/cm^2^), and static conditions for 24 h (middle panel), with high-magnification views of the regions outlined in white boxes. Scale bars: 100 μm. The mRNA expression levels of CNR1 in hiPSC-ECs were quantified by qPCR and normalized to those of GAPDH (right panel)

To investigate the roles of CB1 in atherosclerosis, we analyzed gene expression microarray datasets from the GEO. Three gene expression profiles were used: GSE21545, GSE40231, and GSE46401 (Fig. 1A). RNA expression data from atherosclerotic plaque tissue and nonatherosclerotic tissue samples from patients with cardiovascular disease were extracted, and the expression of CNR1 was greater in atherosclerotic tissues than in nonatherosclerotic tissues (Fig. 1B and Figure S1A). Apoe^−/−^ mice are the most widely used murine model for atherosclerosis [34]. Apoe^−/−^ mice fed a high-fat diet typically develop substantial atherosclerotic plaque formation within 12 weeks. An accelerated carotid atherosclerosis model was generated by performing PCAL in Apoe^−/−^ mice [35]. Immunofluorescence staining revealed that endothelial CB1 expression was markedly higher in the ligated carotid artery (atherosclerotic) compared with the non-ligated contralateral carotid artery (non-atherosclerotic) in the PCAL Apoe⁻/⁻ mouse model (Fig. 1C). Atherosclerotic plaque formation in the endothelium is site specific: disturbed blood flow at the lesser curvature of the aortic arch and branch points promotes plaque formation, whereas steady laminar flow at the greater curvature is atheroprotective [36]. En face immunofluorescence staining in Apoe^−/−^ mice revealed higher CB1 expression in the aortic arch compared to the descending thoracic aorta (Fig. 1D). A cone-and-plate flow chamber based on viscometry technology was used to investigate the effect of disturbed flow on CNR1 expression in human vascular endothelial cells [33]. We found that CNR1 expression was greater under disturbed flow conditions than under laminar flow conditions (Fig. 1E). These findings were observed in various types of vascular endothelial cells, including arterial endothelial cells, venous endothelial cells, and human induced pluripotent stem cell-derived endothelial cells (hiPSC-ECs) (Figure S1B-S1D). These results suggest that CB1 is highly expressed in endothelial cells exposed to disturbed flow and may play a role in the progression of atherosclerosis.

### Transcriptional regulation contributes to endothelial CNR1 upregulation in response to disturbed flow

**Fig. 2:**
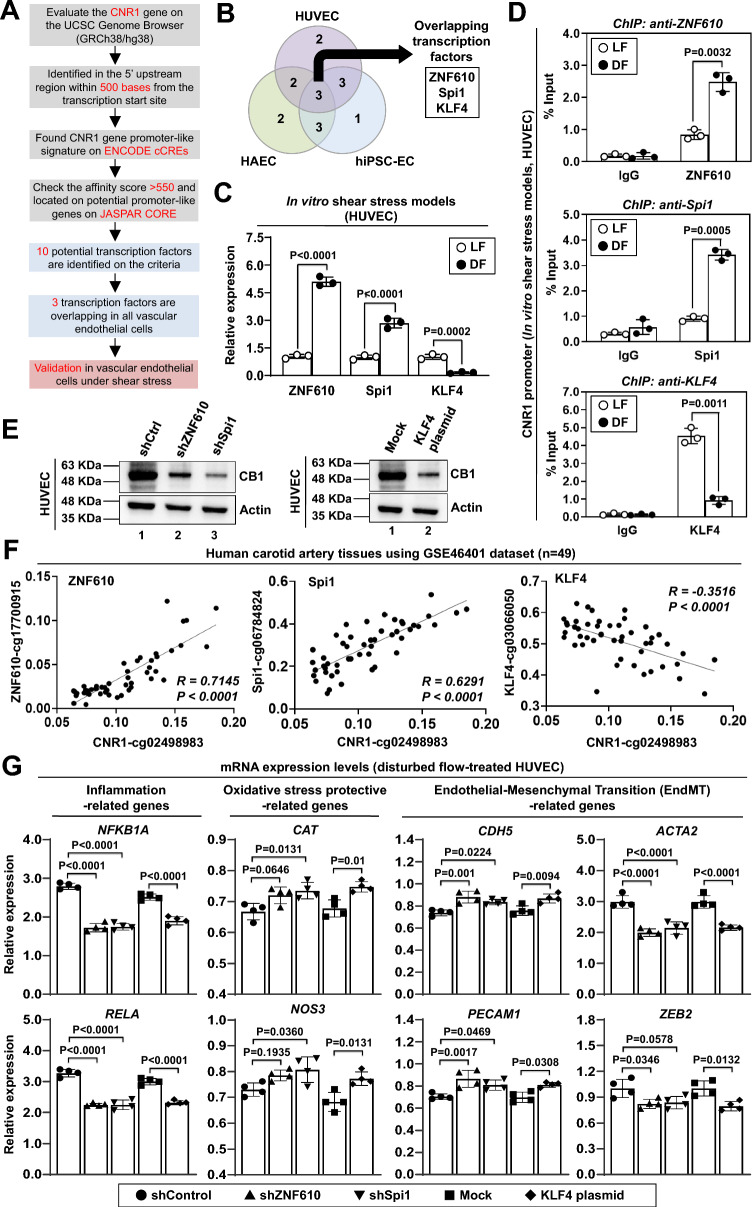
Transcriptional regulation of endothelial CNR1 expression. **A**-**C** Identification of potential transcription factors through analysis of CNR1 promoter sequences. **A** Workflow for screening potential transcription factors involved in CNR1 expression. **B** Venn diagram illustrating the overlap among the ten highest-scoring hits identified via the UCSC Genome Browser (https://genome.ucsc.edu/), which are expressed in all three types of endothelial cells (HUVECs, HAECs, and hiPSC-ECs). **C** The mRNA expression levels of three transcription factors (KLF4, ZNF610, and Spi1) in HUVECs were quantified by qPCR. The mRNA expression levels were normalized to those of GAPDH. **D** Different antibodies were used for ChIP-qPCR to measure the binding of ZNF610, Spi1, and KLF4 to the CNR1 promoter in HUVECs under shear stress. Data were analyzed and are plotted as percent (%) of input DNA. **E** Roles of ZNF610, Spi1, and KLF4 in regulating CB1 expression and endothelial functions. HUVECs were transfected with shRNA to knock down ZNF610 or Spi1 or with a plasmid to overexpress KLF4, followed by exposure to disturbed flow for 24 h. Total cell lysates were analyzed by Western blotting using the indicated antibodies. **F** Correlations between CNR1 and three transcription factors in human carotid arteries according to the GSE46401 dataset. **G** Roles of ZNF610, Spi1, and KLF4 in regulating CNR1 expression and endothelial functions. The expression of inflammation-related genes, oxidative stress protection-related genes, and endothelial–mesenchymal transition (EndMT)-related genes was measured by qPCR. mRNA expression was normalized to GAPDH expression

Since CNR1 mRNA expression was found to be elevated in atherosclerotic lesions and disturbed flow-treated endothelial cells, we further investigated the mechanism underlying disturbed flow-induced CNR1 mRNA expression in endothelial cells. The UCSC Genome Browser database identified 10 transcription factor-binding sites (TFBSs) in the CNR1 gene promoter (Fig. 2A). We found that exposure to disturbed flow upregulated the mRNA expression of ZNF610 and Spi1 but downregulated the mRNA expression of KLF4 in arterial endothelial cells, venous endothelial cells, and hiPSC-ECs (Figs. 2B, 2C, and S2A). The ChIP-qPCR assay revealed that disturbed flow promoted the binding of ZNF610 and Spi1 to the CNR1 promoter, while KLF4 dissociated from the promoter, ultimately leading to increased CNR1 transcriptional activation in endothelial cells (Fig. 2D). We found that CNR1 expression was inhibited by either ZNF610 or Spi1 knockout (KO), as well as by the overexpression of KLF4 in endothelial cells (Fig. 2E). With GEO datasets, a positive correlation between ZNF610 and Spi1 expression with CNR1 expression was observed in human carotid artery tissues (Fig. 2F). In contrast, a negative correlation was observed between KLF4 and CNR1 expression. The CNR1 receptor normally associates with Gi signaling, resulting in the inhibition of cAMP production. We found that knocking out either ZNF610 or Spi1 or overexpressing KLF4 reversed the impaired cAMP levels induced by disturbed flow in HUVECs (Figure S2B). These results confirm that the transcription factors ZNF610, Spi1, and KLF4 contribute to the transcriptional regulation of endothelial CNR1 upregulation under disturbed flow conditions.

CB1 receptor activation in endothelial cells may amplify the MAPK activation-cell death pathway under pathological conditions through excessive inflammation and oxidative stress, thereby contributing to the development of endothelial dysfunction and the pathophysiology of cardiovascular diseases [37, 38]; therefore, we investigated the expression of these genes in HUVECs exposed to disturbed flow to elucidate the transcriptional regulation of these three transcription factors. We observed that either ZNF610 or Spi1 KO reduced the expression of inflammation-related genes and upregulated oxidative stress protective genes in HUVECs (Fig. 2G). Similarly, overexpression of KLF4 had a comparable effect. Endothelial-to-mesenchymal transition (EndMT) is a dynamic process in which endothelial cells acquire mesenchymal properties and, in turn, contribute to tissue remodeling and growth [39]. EndMT contributes to atherosclerosis by inducing a number of phenotypes ranging from endothelial cell dysfunction to plaque formation [40, 41]. We found that knocking out either ZNF610 or Spi1 or overexpressing KLF4 reduced the expression of mesenchymal markers such as ACTA2 and ZEB2 but increased the expression of endothelial markers such as CDH5 and PECAM1 (Fig. 2G). Monocyte adhesion to endothelial cells is a key event in the development of atherosclerosis [42]. We examined monocyte adhesion to HUVECs under disturbed flow conditions and found that knocking out either ZNF610 or Spi1 or overexpressing KLF4 reduced monocyte adhesion to HUVECs (Figure S2C). In vascular endothelial cells, NF-κB plays an important role in inducing the expression of proinflammatory cytokines, chemotactic factors, and adhesion molecules, thereby promoting monocyte recruitment and atherosclerosis progression [43]. The phosphorylation of NF-κB was reduced in HUVECs by knocking out either ZNF610 or Spi1 or overexpressing KLF4 (Figure S2D). Knocking out either ZNF610 or Spi1 or overexpressing KLF4 also rescued the impaired tube formation induced by disturbed flow in HUVECs (Figure S2E). These results suggest that transcriptional regulation plays a key role in CNR1 upregulation in endothelial cells exposed to disturbed flow.

### Genistein and daidzein bind to the CB1 receptor and inhibit CB1 activity in endothelial cells

**Fig. 3:**
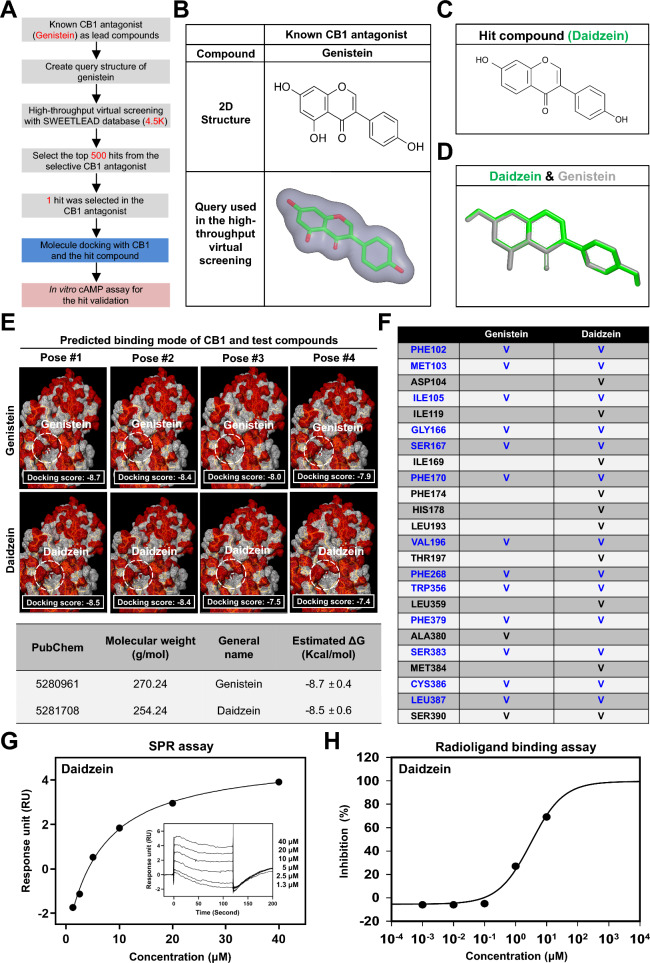
Identification of a cannabinoid receptor 1 (CB1) inhibitor by high-throughput virtual screening. **A** High-throughput virtual screening workflow for lead compound identification. **B** The query structure of a known CB1 antagonist (genistein) was used in the ligand-based high-throughput virtual screening. **C** Molecular structure of the hit compound daidzein. **D** Daidzein shares structural homology with the new CB1 antagonist genistein in ligand-based virtual screening. **E** Docking of the new CB1 antagonists genistein and daidzein into the CB1 receptor. The best fit of genistein and daidzein into CB1 is shown (upper panel). The binding energy of genistein and daidzein was estimated via Mcule molecular docking software (lower panel). **F** Overlapping binding sites of four predicted binding modes for genistein and daidzein within the CB1 receptor are shown in blue. **(G)** Direct interaction between CB1 and daidzein by SPR in vitro assay. Sensorgram of SPR for evaluating the binding affinity between CB1 and daidzein (equilibrium dissociation rate constant; KD = 6.6 μM). **H** Radioligand binding assay of daidzein for the CB1 receptor. The half-maximal inhibitory concentration (IC_50_) was determined using non-linear least squares regression analysis (IC_50_ = 3.78 μM). The inhibition constant (Ki) was calculated using the Cheng-Prusoff equation (Ki = 3.40 μM), based on the observed IC_50_ value and the concentration of radioligand used in the assay

Our previous study revealed that Δ^9^-THC induces vascular inflammation and oxidative stress in endothelial cells via a CB1-mediated pathway, and these effects can be abolished by the CB1 antagonist genistein [32]. To identify new CB1 antagonists, we performed a combined strategy of ligand-based and structure-based virtual screening (Fig. 3A). ROCS software was used for high-throughput virtual screening of the SWEETLEAD cheminformatic database as the chemical library (Fig. 3A and B). We discovered that daidzein, a natural soybean flavonoid, adopted a similar shape to the query structure of the CB1 antagonist genistein (Fig. 3C and D). To refine our findings, a molecular docking analysis was performed to probe the interactions between daidzein and the CB1 receptor, with the known CB1 antagonist genistein used as a positive control. We discovered that daidzein could bind to the CB1 receptor (Fig. 3E). Computational analysis of ligand-receptor interactions revealed that, similar to genistein, daidzein docked into a similar binding pocket of the CB1 protein (Figs. 3F, S3A, and S3B).

To validate the binding ability of daidzein, SPR biosensor analysis was performed. Sensorgram analysis revealed direct binding of daidzein to CB1, with an estimated dissociation constant (KD) of 6.6 μM, indicating moderate affinity (Fig. 3G). Radioligand binding assays further demonstrated that daidzein bound to human CB1, with an IC_50_ of 3.78 μM and an inhibition constant (Ki) of 3.40 μM, whereas no measurable activity toward CB2 was detected (Fig. 3H and Table S3). Rimonabant was included as a positive control. These results demonstrate that daidzein binds to CB1 and exhibits anti-CB1 activity.

### Isoflavone monophosphates reverse disturbed flow-induced oxidative stress, inflammation, and endothelial-to-mesenchymal transition in endothelial cells

Two major isoflavones found in soybean are genistein and daidzein, which are glycoside conjugates of genistein and daidzein, respectively [44]. Natural isoflavones can be classified into four categories: malonyl glucosides, acetyl glucosides, glucosides, and aglycones. Among these forms, the aglycone forms are considered the most bioactive but represent only a minor fraction (< 10%) of the total isoflavone concentration [45]. Although these isoflavones possess pharmacological activity, they have poor water solubility and low gastrointestinal permeability (classified as class IV substances in the Biopharmaceutical Classification System) [46, 47]. These disadvantages lead to poor oral bioavailability of isoflavones and pose major challenges for translational applications.

**Fig. 4:**
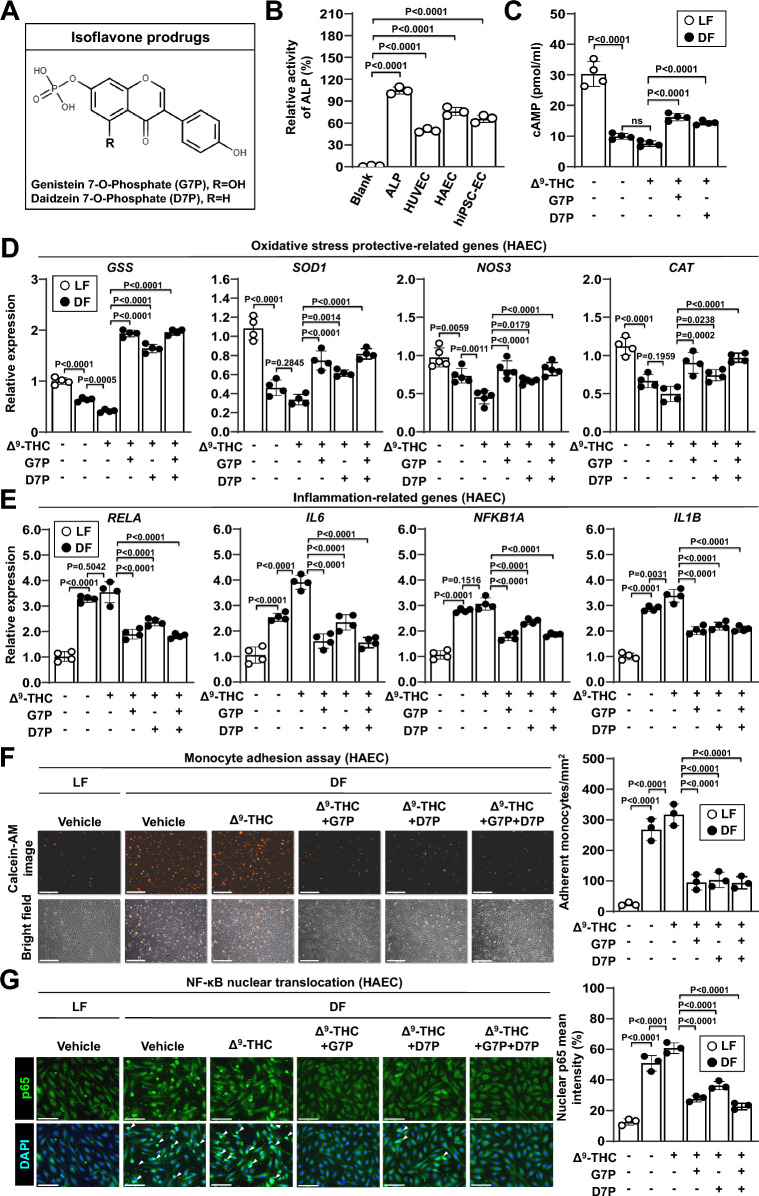
Effects of isoflavone monophosphates on inflammatory response and oxidative stress in endothelial cells. **A** Molecular structures of the isoflavone prodrugs genistein 7-O-phosphate (G7P) and daidzein 7-O-phosphate (D7P) are shown. **B** Relative alkaline phosphatase (ALP) activities in cell lysates from endothelial cells are shown. ALP levels were measured using an ALP activity assay kit and normalized to the ALP-positive control. **(C)** HUVECs were treated with 5 μM Δ^9^-THC, 2 μM G7P, 2 μM D7P, or a combination of G7P and D7P for 24 h under laminar flow (LF) or disturbed flow (DF) conditions. Intracellular cAMP levels are shown. ns, no significance. **D**-**G** HAECs were treated with 5 μM Δ^9^-THC, 2 μM G7P, 2 μM D7P, or a combination of G7P and D7P for 24 h under laminar flow (LF) or disturbed flow (DF) conditions. **D** mRNA expression levels were quantified by qPCR and normalized to GAPDH expression. The mRNA expression levels of CNR1 (left panel) and oxidative stress-related protective genes (right panel) in HAECs are shown. **E** mRNA expression levels of inflammation-related genes in HAECs are shown. **F** Monocyte adhesion assays were performed on disturbed flow-treated HAECs. Fluorescence images (upper left panel) and bright-field images (lower left panel) of THP-1 adhesion were captured using a fluorescence microscopy. Scale bars: 250 μm. The fluorescence intensity of adherent THP-1 monocytes was quantified (right panel). **(G)** Immunofluorescence staining for p65 (Alexa Fluor 488, green fluorescence) and nuclei (DAPI, blue fluorescence). Merged images are shown (left panel). The white arrowhead indicates the nuclear translocation of p65. Scale bar: 150 μm. The quantification of nuclear p65 fluorescence intensity in HAECs was performed using ImageJ software (right panel)

Phosphate prodrugs are generally freely soluble in water and readily hydrolyzed in vivo by ALP, an enzyme widely distributed in a variety of tissues [48]. In our previous work, we generated isoflavone derivatives such as genistein 7-O-phosphate (G7P) and daidzein 7-O-phosphate (D7P) through a biotransformation process [49]. Following oral administration, G7P exhibited the highest AUC₀–∞ for genistein, which was 3.7 and 3.0 times higher than that of aqueous genistein suspension and genistein solution, respectively. Furthermore, LC–MS analysis revealed that oral G7P administration in rats resulted in plasma genistein concentrations of approximately 1–2 μM. These biotransformation products showed high aqueous solubility and were dephosphorylated into their parent isoflavone forms by ALP in Caco-2 cells, consistent with the phosphate prodrug strategy (Figures S4A, S4B, and 4A). G7P and D7P were identified as the major phosphorylation products following B. subtilis-mediated biotransformation, while the corresponding 4’-O-phosphate isomers were detected as minor metabolites (Figure S4C, upper and middle panels). After purification, analytical RP-HPLC confirmed high purity of G7P (98.5%) and D7P (95.2%), with no detectable residual genistein or daidzein, confirming the identity and suitability of these compounds for subsequent experiments (Figure S4C, lower panel). In addition, we found that various vascular endothelial cells also exhibited ALP activity (Fig. 4B). We further examined cAMP levels, MAPK phosphorylation, and β-arrestin expression in endothelial cells exposed to Δ^9^-THC and isoflavone phosphatases under shear stress. Both G7P and D7P (2 μM) reversed the Δ^9^-THC-induced reduction of cAMP in HUVECs under disturbed flow (Fig. 4C). Δ^9^-THC also increased ERK1/2 phosphorylation, which was modestly attenuated by co-treatment with isoflavone phosphatases (Figure S4D). In contrast, β-arrestin expression remained unchanged following exposure to either Δ^9^-THC alone or in combination with isoflavone phosphates (Figure S4E). These results suggest that G7P and D7P can be dephosphorylated into genistein and daidzein, thereby exerting vascular protective effects in endothelial cells.

We further investigated the effects of isoflavone monophsophates on oxidative stress and inflammation in endothelial cells. G7P, D7P, and their combination reversed the disturbed flow-induced changes in the mRNA expression of oxidative stress protection-related genes and inflammation-related genes in various endothelial cells (Figs. 4D, E, and S5A-S5D). Similar effects were observed in endothelial cells treated with the CB1 antagonist rimonabant as a positive control (Figure S5E and S5F). G7P, D7P, and their combination also reduced the expression of endothelial inflammatory adhesion molecules, including VCAM1 and ICAM1, under disturbed flow (Figure S5G). Notably, treatment with isoflavones did not alter CNR1 expression levels (Figure S5H and S5I). Given the attenuated inflammatory profile, we postulated that endothelial cells treated with isoflavone monophosphates are more likely to remain quiescent without contributing to the pathogenesis of atherosclerosis. We tested this hypothesis using monocyte adhesion assays. G7P, D7P, and their combination reversed disturbed flow-induced monocyte adhesion to endothelial cells but also attenuated NF-κB activation (Fig. 4F and G).

**Fig. 5:**
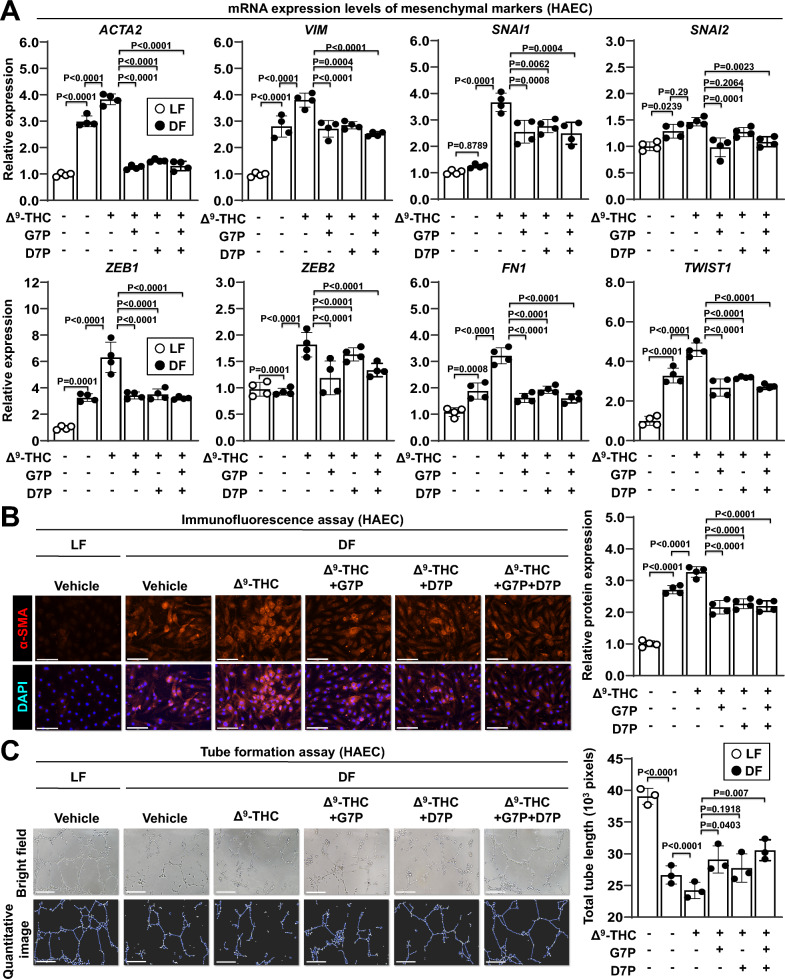
Effects of isoflavone monophosphates on endothelial-mesenchymal transition (EndMT) in endothelial cells. HAECs were treated with 5 μM Δ^9^-THC, 2 μM G7P, 2 μM D7P, or a combination of G7P and D7P for 24 h under laminar flow (LF) or disturbed flow (DF) conditions. **A** mRNA expression levels were quantified by qPCR and normalized to GAPDH expression. The mRNA expression levels of mesenchymal markers in HAECs are shown. **B** Immunofluorescence staining for α-SMA (Cy3, red fluorescence) and nuclei (DAPI, blue fluorescence). Merged images are shown (left panel). Scale bar: 150 μm. The quantification of α-SMA fluorescence intensity in HAECs was performed using ImageJ software (right panel). **C** Tube formation assays were performed on disturbed flow-treated HAECs. Representative images of endothelial tube formation (upper left panel) and quantification images (lower left panel) are shown. Scale bar: 150 μm. The results of the quantitative tube formation assay are presented (right panel)

EndMT is characterized by multiple morphological and physiological changes, including loss of endothelial cell polarity and disruption of intercellular junctions [50]. Characteristic morphological changes were observed in endothelial cells under disturbed flow conditions (Figure S6A). Therefore, we analyzed EndMT markers after treatment with isoflavone monophosphates. Increased mRNA expression of mesenchymal markers and decreased mRNA expression of endothelial markers were detected in endothelial cells under disturbed flow conditions, whereas these effects were reversed by treatment with G7P, D7P, or their combination (Figs. 5A and S6B). Immunofluorescence staining showed that disturbed flow exposure induced the expression of the mesenchymal marker α-SMA and reduced the expression of the endothelial marker CD31, while these effects were reversed by treatment with G7P, D7P, or their combination (Fig. 5B and S6C). Furthermore, treatment with G7P, D7P, or their combination reversed the impaired tube formation induced by disturbed flow in endothelial cells (Fig. 5C and S6D). These findings indicate that isoflavone monophosphates effectively counteract disturbed flow-induced endothelial dysfunction and EndMT in endothelial cells.

### Isoflavone monophosphates attenuate atherosclerosis in acute and chronic mouse models

**Fig. 6:**
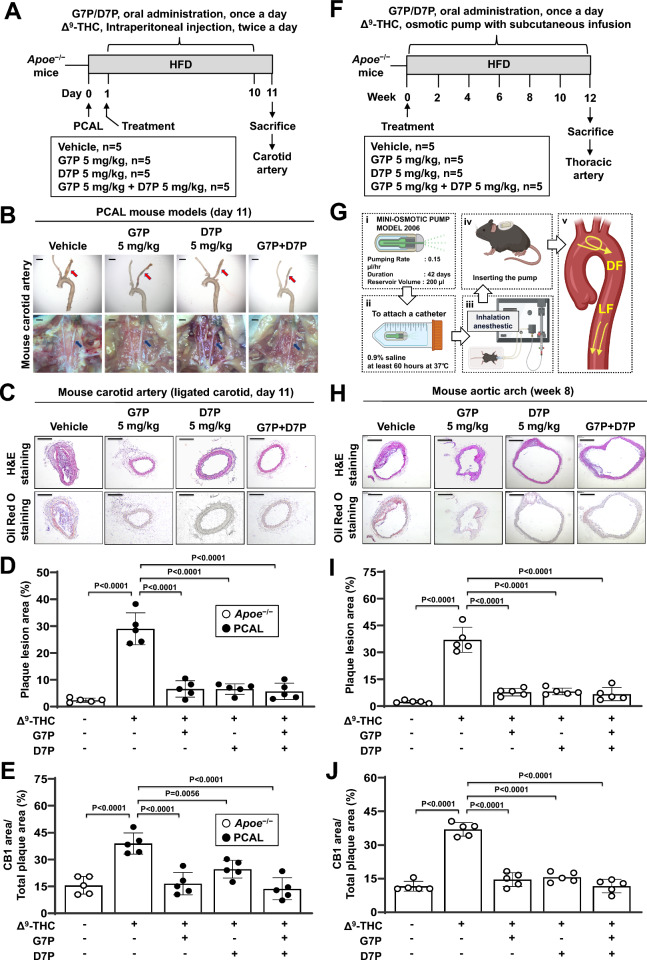
Effects of isoflavone monophosphates on the progression of atherosclerotic lesions in mice. **A**-**E** Partial carotid artery ligation (PCAL) was performed in Apoe^−/−^ mice (8 weeks old). After PCAL, the Apoe^−/−^ mice were divided into four groups: (1) vehicle control (n = 5), (2) G7P (5 mg/kg; n = 5), (3) D7P (5 mg/kg; n = 5), and (4) G7P + D7P (n = 5). All the groups received Δ^9^-THC (1 mg/kg) via intraperitoneal injection twice daily for 10 days. Throughout the 12-day treatment period, the mice were maintained on a high-fat diet (HFD). At the end of the study, the mice were euthanized. **A** Schematic overview of the experimental design. **B** Gross images of mouse carotid arteries are shown. The arrowhead indicates the ligated carotid artery. The scale bar represents 1 mm. **C** Carotid artery sections were counterstained with hematoxylin and eosin (H&E), and a representative slide is presented with scale bars at 275 μm (upper panel). Oil red O staining of atherosclerotic plaques in cross-sections of the mouse carotid artery (lower panel); scale bar, 275 μm. **D** Atherosclerotic plaques were quantified. **E** Quantification of the CB1-positive area within carotid artery sections was performed. **F**-**J** Apoe^−/−^ mice (8 weeks old) were divided into four groups: (1) vehicle control (n = 5), (2) G7P (5 mg/kg; n = 5), (3) D7P (5 mg/kg; n = 5), and (4) G7P + D7P (n = 5) groups. The mice were administered Δ^9^-THC (1 mg/kg/day) subcutaneously via osmotic pumps, along with daily oral administration of G7P or D7P. All the groups were fed an HFD throughout the 12-week treatment period. At the end of the study, the mice were euthanized. **F** Schematic overview of the experimental design. **G** Δ^9^-THC (1 mg/kg/day) was administered subcutaneously using osmotic pumps to exacerbate endothelial dysfunction in Apoe^−/−^ mice. Disturbed flow, DF; Laminar flow, LF. **H** Aortic arch sections were counterstained with hematoxylin and eosin (H&E), and a representative slide is presented (upper panel). Oil red O staining of atherosclerotic plaques in cross-sections of the mouse aortic arch (lower panel). Scale bar: 50 μm. **I** Atherosclerotic plaques were quantified. **J** Quantification of the CB1-positive area within aortic arch sections was performed

An Apoe^−/−^ mouse model was used to investigate the effects of isoflavone monophosphates on atherosclerosis. PCAL was performed in Apoe^−/−^ mice at 12 weeks. After PCAL, the Apoe^−/−^ mice were divided into four groups: (1) vehicle control, (2) G7P, (3) D7P, or (4) G7P and D7P (n = 5/group). All the mice were fed an HFD and administered Δ^9^-THC via intraperitoneal injection for 10 days (Fig. 6A). At the end of the treatment protocol, the Apoe^−/−^ mice were euthanized and subjected to histological analysis. Compared with the unligated control arterial vessel, the ligated carotid artery showed increased fat deposition by Oil Red O staining and neointimal thickening by H&E staining (Fig. 6B and C). Δ^9^-THC further increased plaque area, whereas treatment with G7P, D7P, or their combination reduced lesion size (Fig. 6C and D). CB1 expression in the endothelium of the ligated carotid artery was analyzed using aortic plaques stained with anti-CD144 and anti-CB1 antibodies. Immunofluorescence analysis revealed that the CB1-positive area was significantly increased by Δ^9^-THC administration and decreased by treatment with G7P, D7P, or their combination (Figure S7A and 6E). In parallel, the mRNA expression of endothelial-specific inflammatory adhesion genes VCAM1 and ICAM1 in carotid artery tissues was reduced by all three treatment groups (Figure S7B).

To evaluate the therapeutic window of G7P and D7P, we tested a lower dose (0.5 mg/kg) in PCAL mouse models, with the CB1 antagonist rimonabant included as a positive control (Figure S8A). At this dose, G7P, D7P, or their combination resulted in only modest reductions in atherosclerosis, whereas rimonabant significantly reduced plaque formation (Figure S8B-S8D). These findings support a role of CB1 antagonism in reducing atherosclerosis and suggest that the effective dose range for G7P and D7P in mice is approximately 0.5–5 mg/kg. Further dose-ranging studies are needed to determine the optimal therapeutic dosage.

To investigate the effects of isoflavone monophophates on chronic atherosclerosis progression, we fed Apoe^−/−^ mice an HFD and administered Δ^9^-THC via osmotic pumps for 12 weeks (Fig. 6F and G). The mice were treated with (1) vehicle control, (2) G7P, (3) D7P, or (4) G7P and D7P (n = 5/group). After 12 weeks of an HFD, the mice were euthanized, and the atherosclerotic lesion area was examined by cross-sections of the aortic root and en face analysis of the thoracic aorta. Cross-sectional analysis revealed that Δ^9^-THC treatment significantly increased plaque size, whereas treatment with G7P, D7P, or their combination attenuated plaque formation, as shown by oil red O staining (Fig. 6H and I). CB1 expression in the endothelium of the aortic root was analyzed using aortic plaques stained with anti-CD144 and anti-CB1 antibodies. The CB1-positive area was also increased by Δ^9^-THC administration and rescued by treatment with G7P, D7P, or their combination (Figures S9 and 6 J). Importantly, no mortality occurred in any treatment group during the 12-week daily oral gavage period, and no animals were excluded from the study. These results suggest that Δ^9^-THC exacerbated atherosclerosis formation in atherosclerotic plaques, which was ameliorated by treatment with isoflavone monophosphates through the inhibition of endothelial CB1.

## Discussion

Atherosclerosis is fundamentally an endothelial disease driven by hemodynamic forces. Decades of vascular biology research have established that flow patterns critically regulate endothelial phenotype: laminar shear stress promotes quiescence and vascular homeostasis, whereas disturbed flow induces oxidative stress, inflammation, EndMT, and lesion-prone endothelial states that initiate and accelerate plaque formation [51, 52]. Mechanical forces not only modulate classical mechanosensors but also orchestrate complex transcriptional networks that determine vascular health and disease [53, 54]. Despite extensive knowledge regarding flow-mediated signaling pathways, the contribution of endothelial cannabinoid signaling to flow-responsive vascular dysfunction has remained largely unexplored. Our study identifies mechanosensitive CB1 as a previously unrecognized regulator linking disturbed flow to endothelial dysfunction and atherosclerosis. We demonstrate that disturbed flow activates endothelial CB1 and further enhances its transcription through a coordinated mechanism involving ZNF610 and Spi1 binding to the CNR1 promoter and dissociation of the atheroprotective transcription factor KLF4. These findings expand the current mechanobiology framework by positioning CB1 not only as a mediator of metabolic and inflammatory signaling but also as a flow-responsive molecular switch that amplifies atherogenic endothelial phenotypes. Building on this mechanistic insight and the causal role of CB1 activation, we developed water-soluble, orally bioavailable isoflavone monophosphate prodrugs (G7P and D7P) that function as endothelial-targeted CB1 antagonists. These compounds robustly attenuated disturbed-flow-induced inflammation, oxidative stress, and monocyte adhesion in human endothelial cells, and they reduced atherosclerotic lesion formation in both acute and chronic Apoe⁻/⁻ mouse models. Together, our findings position endothelial CB1 within the broader landscape of atherosclerosis research and demonstrate that isoflavone phosphates ameliorate disturbed flow-induced endothelial dysfunction in vitro and attenuate atherosclerosis in vivo, indicating their potential translational value.

Chemical synthesis of monophosphate derivatives of flavonoids often requires protection-deprotection steps and harsh reaction conditions, and is associated with poor regioselectivity, frequently generating side products such as oligophosphate esters [55]. Although G7P and D7P can be chemically synthesized, we intentionally selected a biotransformation-based production strategy for both scientific and translational reasons. Bacillus subtilis-mediated biotransformation converts soybean-derived substrates into phosphorylated isoflavones through an enzymatic, nature-mimicking pathway that avoids harsh chemical reagents. This enzymatic process offers high substrate specificity and generates fewer unwanted by-products compared with multi-step chemical synthesis. Moreover, when coupled with downstream purification and analytical validation, this strategy yields highly pure compounds suitable for biological investigation. Importantly, the biotransformation approach aligns with our long-term goal of developing safe, fermentation-derived CB1-modulating agents for preventing endothelial dysfunction and enables robust evaluation of the vascular protective effects of G7P and D7P.

Many studies have reported beneficial effects of genistein and daidzein on human health [56, 57], motivating us to study these soy-derived isoflavones. However, according to the BCS, both compounds belong to class IV, meaning they have low solubility, poor gastrointestinal permeability, and low bioavailability. To overcome this, various chemical, enzymatic, and microbial strategies have been used to improve their solubility and usability [58–60]. Microbial phosphorylation of isoflavones is rarely studied, with only two reports in the past decade [61, 62]. Kanakubo et al. produced water-soluble isoflavone analogs using Bacillus subtilis NCI-21011, initially thought to be sulfate esters but later found to be phosphates [63]. More recently, Hsu et al. demonstrated that B. subtilis var. natto BCRC 80517 can bioconvert genistein and daidzein into their 7-O-phosphate forms, which are subsequently dephosphorylated by intestinal ALP on enterocytes [31]. This was demonstrated by comparing the phosphate removal efficiency of G7P with that of fosphenytoin, an FDA-approved phosphate prodrug [64]. To evaluate G7P and D7P as vascular prodrugs, we assessed ALP activity in various endothelial cells and confirmed their conversion to genistein and daidzein. To determine whether isoflavone phosphates exert protective effects independent of cannabis exposure, HUVECs were subjected to disturbed flow without Δ^9^-THC treatment. Both G7P and D7P significantly reduced inflammation, oxidative stress, and EndMT under these conditions (Figure S10). These results indicate that the vascular protective actions of isoflavone phosphates are not limited to Δ^9^-THC-related endothelial dysfunction and may also be relevant for individuals who do not use cannabis. Further in vivo studies will be needed to confirm Δ^9^-THC-independent protective effects in atherosclerosis. In addition, these isoflavone phosphates were evaluated in healthy volunteers through oral administration at National Taiwan University Hospital, where they were found to significantly enhance the oral bioavailability of the parent isoflavones.

Cannabinoid-induced behaviors such as hypolocomotion, analgesia, hypothermia, and catalepsy are known as the Billy Martin tetrad [65, 66]. These effects are mainly mediated by central CB1 activation, and some, like sedation and analgesia, may have therapeutic value [67]. In our previous study, we assessed the neurobehavioral effects of isoflavones in C57BL/6 J mice using standard assays: activity chamber for locomotion, hot plate test for analgesia, rectal thermometer for hypothermia, and bar test for catalepsy [65]. Δ^9^-THC significantly reduced locomotion and induced analgesia, hypothermia, and catalepsy. Genistein alone had no effect on these behaviors and did not reduce the effects of Δ^9^-THC when co-administered [32]. Biodistribution analysis showed that genistein accumulated in abdominal organs, thoracic aorta, heart, and lungs, but was barely detectable in the brain, consistent with previous reports of poor BBB penetration [68]. These findings suggest that soy isoflavones may block the harmful peripheral cardiovascular effects of cannabinoid-induced endothelial CB1 activation while preserving beneficial central effects such as sedation and analgesia.

Rimonabant was the first CB1 antagonist approved by the European Medicines Agency for the treatment of obesity, but it was later withdrawn from the market due to severe psychiatric side effects, including depression, anxiety, and suicidal ideation [69]. To mitigate these CNS adverse effects, pharmaceutical companies have developed peripherally restricted CB1 antagonists by modifying the chemical structure of rimonabant or conjugating bulky groups to reduce BBB penetration [70]. For example, INV-202 (MRI-1891), a peripherally restricted CB1 antagonist developed by Novo Nordisk, is currently in phase 2 clinical trials [71]. It shows a reduced brain-to-plasma ratio (7%) compared to rimonabant (100%) and ibipinabant (22%) [72]. Although INV-202 and other rimonabant-derived compounds have reduced brain penetration, their partial brain exposure may still lead to CNS-related side effects. In contrast to rimonabant-derived compounds, soy isoflavones such as genistein offer a different approach. Several studies have shown that genistein has poor BBB permeability [68]. Consistent with these findings, our previous study demonstrated that genistein does not cross the BBB in mice or interfere with cannabinoid-mediated central effects. These properties make genistein a promising lead compound for the development of novel peripherally restricted CB1 antagonists. Using ligand-based virtual screening, we further identified daidzein, another natural isoflavone enriched in soy, which also exhibited anti-CB1 activity and vascular protective effects. However, compared with rimonabant (active in the nanomolar range), daidzein shows only micromolar activity and needs further optimization.

**Fig. 7:**
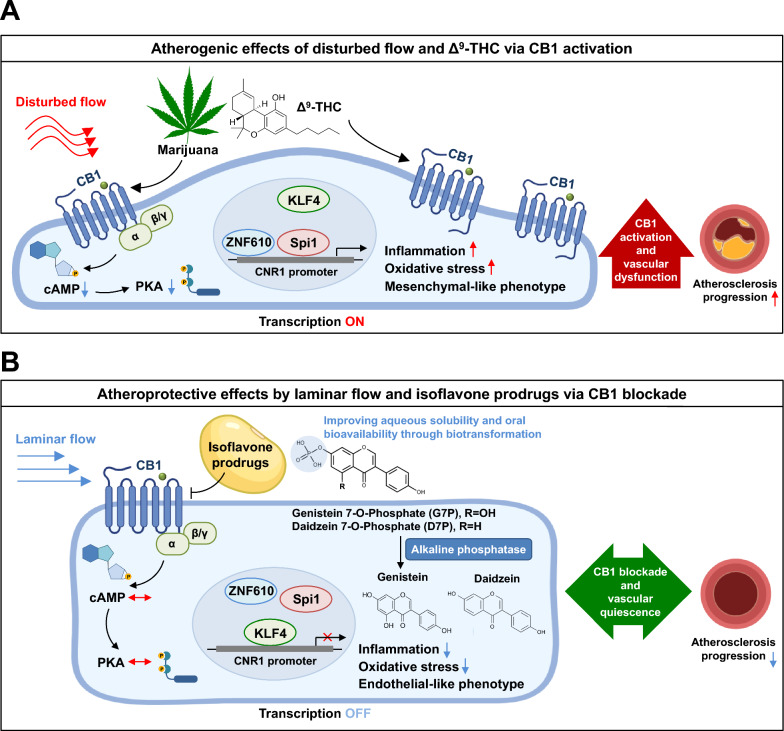
Schematic of the targeting of endothelial CB1 by water-soluble isoflavone monophosphates to ameliorate disturbed flow-induced atherosclerotic endothelial dysfunction. Endothelial CB1 expression is elevated in atherosclerotic lesions, and its upregulation in response to disturbed flow is driven by transcriptional regulation involving KLF4, Spi1, and ZNF610. The soybean isoflavones genistein and daidzein act as CB1 antagonists to inhibit endothelial dysfunction. To improve the water solubility and oral bioavailability of genistein and daidzein, we developed their monophosphate prodrugs, genistein 7-O-phosphate (G7P) and daidzein 7-O-phosphate (D7P), using a biotransformation-based strategy. Pharmacological inhibition of CB1 with isoflavone monophosphates effectively reversed endothelial inflammation, oxidative stress, and endothelial-to-mesenchymal transition (EndMT) induced by disturbed flow. Furthermore, oral administration of G7P and D7P significantly reduced atherosclerotic plaque formation in both acute and chronic atherosclerosis mouse models

To investigate whether the vascular protective effects of G7P and D7P are CB1 dependent, CB1-deficient endothelial cells were generated using the CRISPR/Cas9 system (Figure S11A and S11B). Knockout of CB1 in HUVECs reversed Δ^9^-THC-induced endothelial inflammation and oxidative stress (Figure S11C). Likewise, disturbed flow-induced inflammation, oxidative stress, and EndMT were markedly abolished in CB1-deficient cells, further supporting the therapeutic potential of targeting endothelial CB1 (Figure S11D). Since CB1 deficient cells exhibited low levels of inflammation, oxidative stress, and EndMT, comparable to those of normal endothelial cells under laminar flow, it was difficult to detect further reductions in these parameters upon treatment with isoflavone phosphates. Nevertheless, SPR assays and radioligand binding assays confirmed the direct binding of isoflavones to CB1 receptors and their antagonistic effects (Fig. 7).

Multitarget effects are a common feature of small-molecule compounds, including flavonoid derivatives [73]. Studies have reported that flavonoids can bind to estrogen receptors due to their structural similarity to estrogen [74]. They also inhibit the kinase activity of several signal proteins, such as epidermal growth factor receptor (EGFR), ERK1/2, RSK2, MKK4, and Cot in an ATP-competitive manner [75]. To evaluate whether daidzein and genistein would bind to other targets, ligand-based virtual screening for target prediction was performed using the SwissTargetPrediction database [76]. The results showed that genistein may also target proteins such as thromboxane-A synthase, monoamine oxidase A, EGFR, and estrogen receptor (Table S4). Daidzein was predicted to interact with targets including aldehyde dehydrogenase, carbonic anhydrase, monoamine oxidase A, and estrogen receptor (Table S5). While isoflavones exert anti-atherosclerotic effects in part through CB1 inhibition, we cannot exclude the possibility that they also act via other additional mechanisms independent of the CB1-mediated pathway. Epidemiological studies have reported a lower incidence of cardiovascular and coronary heart diseases, including atherosclerosis, in populations with higher soy intake [77, 78]. Genistein and daidzein are the main isoflavones contained in soy-based foods that are regularly consumed in Asian countries [79]. Soy isoflavones are generally recognized as safe dietary supplements [80]. Although they exhibit multitarget effects, their safety profile supports further investigation as potential lead compounds for therapeutic optimization.

This study has several limitations. First, although isoflavone phosphates reduced disturbed-flow-induced endothelial dysfunction in vitro and attenuated atherosclerosis in vivo mouse models, we did not perform functional vascular assays such as wire-myograph studies in Apoe⁻/⁻ mice. These will be essential in future work to validate the translational relevance of these compounds. Second, although G7P and D7P exhibited comparable protective effects in vitro and in vivo, our radioligand binding assays revealed that genistein (IC_50_ = 0.15 μM; Ki = 0.14 μM) exhibits approximately ten-fold stronger CB1 antagonistic activity than daidzein (IC_50_ = 3.78 μM; Ki = 3.40 μM). Additionally, ligand-based virtual screening suggested that genistein and daidzein may interact with distinct protein targets. These findings indicate that CB1 antagonism alone does not fully account for the comparable vascular protective effects of G7P and D7P, and that additional mechanisms may contribute to their actions. Third, only male Apoe⁻/⁻ mice were used in this study. Epidemiological studies indicate that males develop atherosclerosis earlier, whereas the risk in females increases after menopause [81]. Because endothelial CB1 is expressed in both sexes and the protective effects of isoflavone phosphates act through blocking endothelial CB1 activation, we selected male mice to minimize biological variability in vivo. However, sex-dependent differences in vascular biology and atherosclerosis are well-recognized, and studies in female mice will be needed to determine whether similar protective effects occur across sexes. Finally, in both the acute and chronic Apoe⁻/⁻ models, treatment began at the onset of disease induction. Therefore, our experiments primarily evaluate preventive rather than therapeutic effects. Future studies are needed to determine whether isoflavone phosphates can reverse established atherosclerosis.

## Conclusions

Our study demonstrated that disturbed flow activates the CB1-dependent signaling pathway, leading to endothelial dysfunction and atherosclerosis. We identified G7P and D7P, novel CB1 antagonists derived from genistein and daidzein, which effectively mitigate disturbed flow-induced endothelial dysfunction and atherosclerosis. These prodrugs show increased water solubility and bioavailability and require lower dosages than genistein does to achieve their therapeutic effects. Our findings suggest that G7P and D7P could serve as efficient, safer oral CB1 antagonists for treating atherosclerosis.

## Supplementary Information

Additional file1 (PDF 12019 KB)

## Data Availability

The data supporting the findings of this study are available from the corresponding author upon reasonable request.

## Abbreviations

ALP: Alkaline phosphatase
Apoe^−/−^: Apolipoprotein E-deficient
AP-1: Activator protein 1
BSA: Bovine serum albumin
CB1: Cannabinoid receptor 1
CB2: Cannabinoid receptor 2
ChIP‑qPCR: Chromatin IP and quantitative PCR
cAMP: Cyclic adenosine monophosphate
D7P: Daidzein 7-O-phosphate
DAPI: 4ʹ,6-Diamidino-2-phenylindole
Δ^9^-THC: Delta-9-tetrahydrocannabinol
DF: Disturbed flow
EGFR: Epidermal growth factor receptor
ELISA: Enzyme-linked immunosorbent assay
EndMT: Endothelial-mesenchymal transition
G7P: Genistein 7-O-phosphate
GEO: Gene Expression Omnibus
GPCR: G protein-coupled receptor
HAECs: Human aortic endothelial cells
H&E: Hematoxylin and eosin
HFD: High-fat diet
hiPSC-ECs: Human induced pluripotent stem cell-derived endothelial cells
HPLC: High performance liquid chromatography
HUVECs: Human umbilical vein endothelial cells
IACUC: Institutional Animal Care and Use Committee
IC_50_: Half-maximal inhibitory concentration
K_D_: Dissociation constant
Ki: Inhibition constant
KLF4: Krüppel-like factor 4
KO: Knockout
LF: Laminar flow
NB: Nutrient broth
NF-κB: Nuclear factor kappa-light-chain-enhancer of activated B cells
NRF2: Nuclear factor erythroid 2-related factor 2
OCT: Optimal cutting temperature
PFA: Paraformaldehyde
PCAL: Partial carotid artery ligation
RU: Resonance units
qPCR: Real‑time polymerase chain reaction
RP-HPLC: Reverse phase high performance liquid chromatography
SPR: Surface plasmon resonance
TAZ: Transcriptional coactivator with PDZ-binding motif
TEAD: TEA domain transcription factors
YAP: Yes-associated protein
ZNF610: Zinc finger protein 610

## Availability of data and materials

No datasets were generated or analysed during the current study.
