# Supplementary material for: Targeting mechanosensitive cannabinoid receptor 1 with isoflavone prodrugs attenuates atherosclerotic endothelial dysfunction
Source: J Biomed Sci. 2026 Jan 21;33:12. doi: 10.1186/s12929-026-01214-5 (PMC12822132; doi:10.1186/s12929-026-01214-5)
Supplement: Supplementary file 1 — Additional file1 (PDF 12019 KB) [file 12929_2026_1214_MOESM1_ESM.pdf]

Supplementary Information

Targeting mechanosensitive cannabinoid receptor 1 with isoflavone prodrugs attenuates atherosclerotic endothelial dysfunction

Dai-Jung Chung[1], Shao-Peng Chen[1], Wei-Hsuan Liu[1], Chia-Yu Liu[1], Nan-Wei Su[2,3], Chen Hsu[2], Hsin-Ya Tsai[2], Kai-Chien Yang[1, 4], Cho-Kai Wu[4], Sheng-Wei Lin[5], Jiun-Jie Shie[6], Ming-Tao Zhao[7, 8, 9], Tzu-Tang Wei[1, 10]*

Inventory of Supplementary Information

Supplementary Data

Figure S1, related to Figure 1

Figure S2, related to Figure 2

Figure S3, related to Figure 3

Figure S4 and S5, related to Figure 4

Figure S6, related to Figure 5

Figure S7, S8, and S9, related to Figure 6

Figure S10

Figure S11

Table S1

Table S2

Table S3, related to Figure 3

Table S4

Table S5

Supplementary Materials and Methods

Figure S1

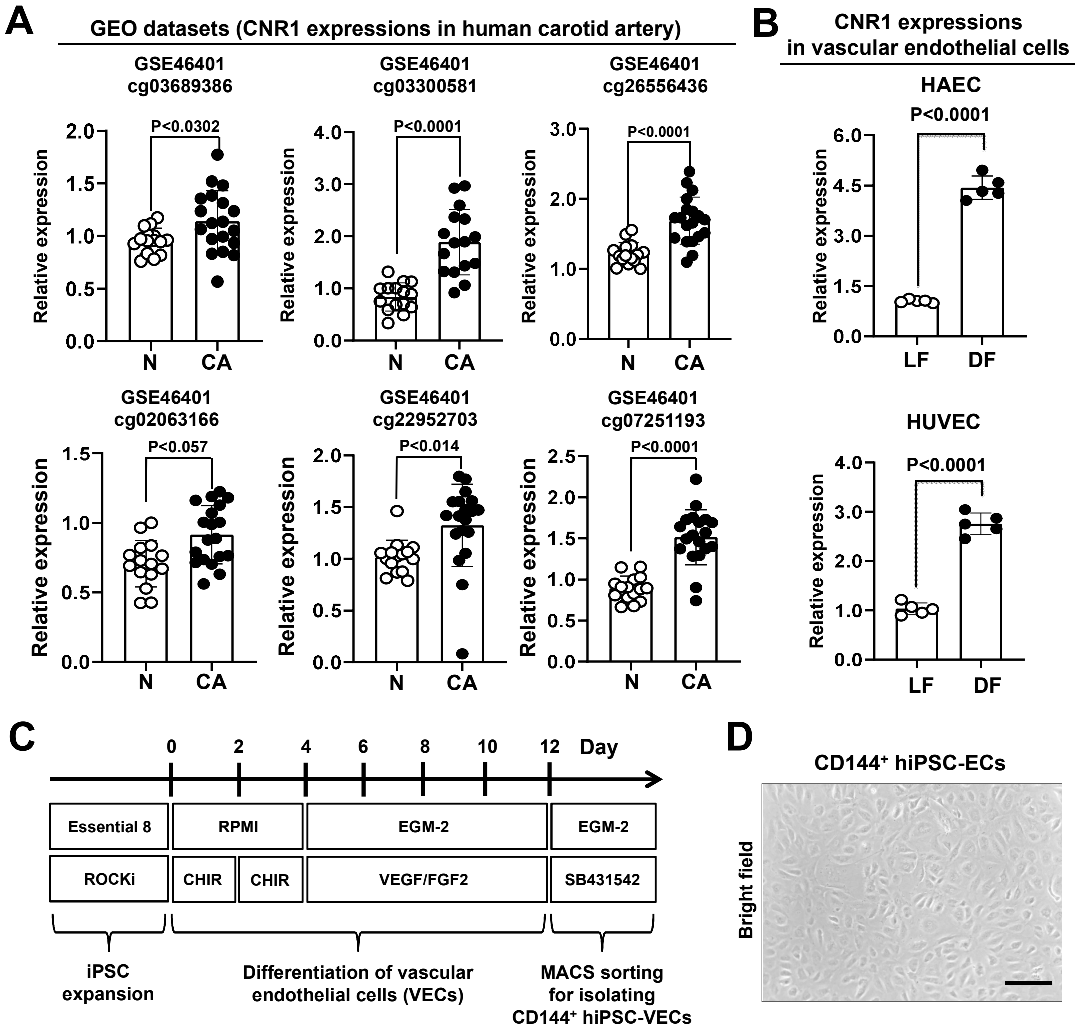

Figure S1. Expression levels of CNR1 in vascular endothelial cells. (A) Microarray data from the GSE46401 dataset, which includes patients with carotid atherosclerotic plaques and carotid artery disease, were obtained from the NCBI GEO database. **(B)** mRNA expression levels of the CNR1 gene in HAEC and HUVEC were quantified by qPCR analysis. GAPDH was used as the normalization control. **(C)** Schematic of the vascular endothelial cell differentiation protocol, achieved through the sequential administration of chemicals and growth factors. **(D)** Representative images of hiPSC-ECs are shown. Scale bars: 50 μm.

Figure S2

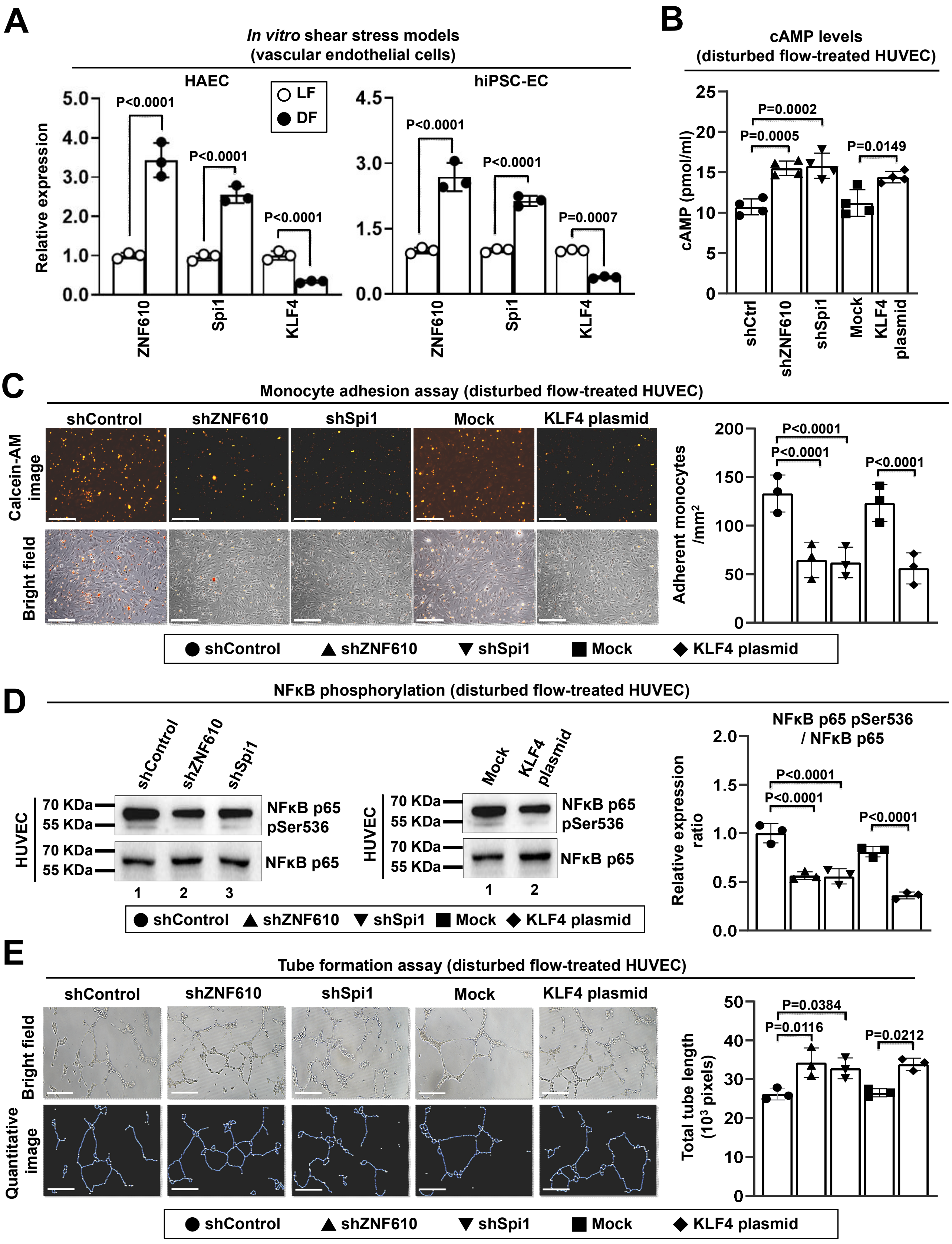
A
In vitro shear stress models
(vascular endothelial cells)
HAEC
hiPSC-EC
LF
DF
Relative expression
P<0.0001
P<0.0001
P<0.0001
P<0.0001
P<0.0001
P=0.0007
ZNF610
Spi1
KLF4
B
cAMP levels
(disturbed flow-treated HUVEC)
cAMP (pmol/ml)
P=0.0002
P=0.0005
P=0.0149
shCtrl
shZNF610
shSpi1
Mock
KLF4 plasmid
C
Monocyte adhesion assay (disturbed flow-treated HUVEC)
shControl
shZNF610
shSpi1
Mock
KLF4 plasmid
Calcein-AM image
Bright field
Adherent monocytes /mm²
P<0.0001
P<0.0001
P<0.0001
shControl
shZNF610
shSpi1
Mock
KLF4 plasmid
D
NFκB phosphorylation (disturbed flow-treated HUVEC)
shControl
shZNF610
shSpi1
Mock
KLF4 plasmid
HUVEC
70 KDa
55 KDa
NFκB p65 pSer536
NFκB p65
1
2
3
NFκB p65 pSer536 / NFκB p65
Relative expression ratio
P<0.0001
P<0.0001
P<0.0001
E
Tube formation assay (disturbed flow-treated HUVEC)
Bright field
Quantitative image
Total tube length (10³ pixels)
P=0.0384
P=0.0116
P=0.0212

Figure S2. Transcriptional regulation of CNR1 expression in endothelial cells under disturbed flow exposure. (A) mRNA expression levels of predicted transcription factor genes (KLF4, Spi1, and ZNF610) involved in regulating CNR1 expression in endothelial cells (HAEC and hiPSC-ECs) under disturbed flow exposure. **(B)** HUVEC were transfected with shZNF610 or shSpi1 to downregulate expression, or with a plasmid to overexpress KLF4, followed by exposure to disturbed flow for 24 hours. cAMP levels are shown. **(C)** The role of ZNF610, Spi1, and KLF4 in regulating monocyte adhesion. Monocyte adhesion assays were performed on disturbed flow-treated HUVEC. Fluorescent images (upper left panel) and bright-field images (lower left panel) of THP-1 adhesion were captured using a fluorescence microscope. Scale bars: 250 μm. The fluorescence intensity of adherent THP-1 monocytes was quantified (right panel). **(D)** HUVEC were transfected with shRNA to knock down ZNF610 or Spi1, or with a plasmid to overexpress KLF4, followed by exposure to disturbed flow for 24 hours. The role of ZNF610, Spi1, and KLF4 in regulating NF-κB activation. Western blot analysis was performed on total cell lysates using indicated antibodies (left panel). Western blot images were quantified by ImageJ software (right panel). **(E)** The role of ZNF610, Spi1, and KLF4 in regulating angiogenic capacity. Tube formation assays were performed on disturbed flow-treated HUVEC. Representative images of endothelial tube formation (upper left panel) and quantification images (lower left panel) are shown. Scale bar: 150 μm. Quantitative results of the tube formation assay are presented (right panel).

Figure S3

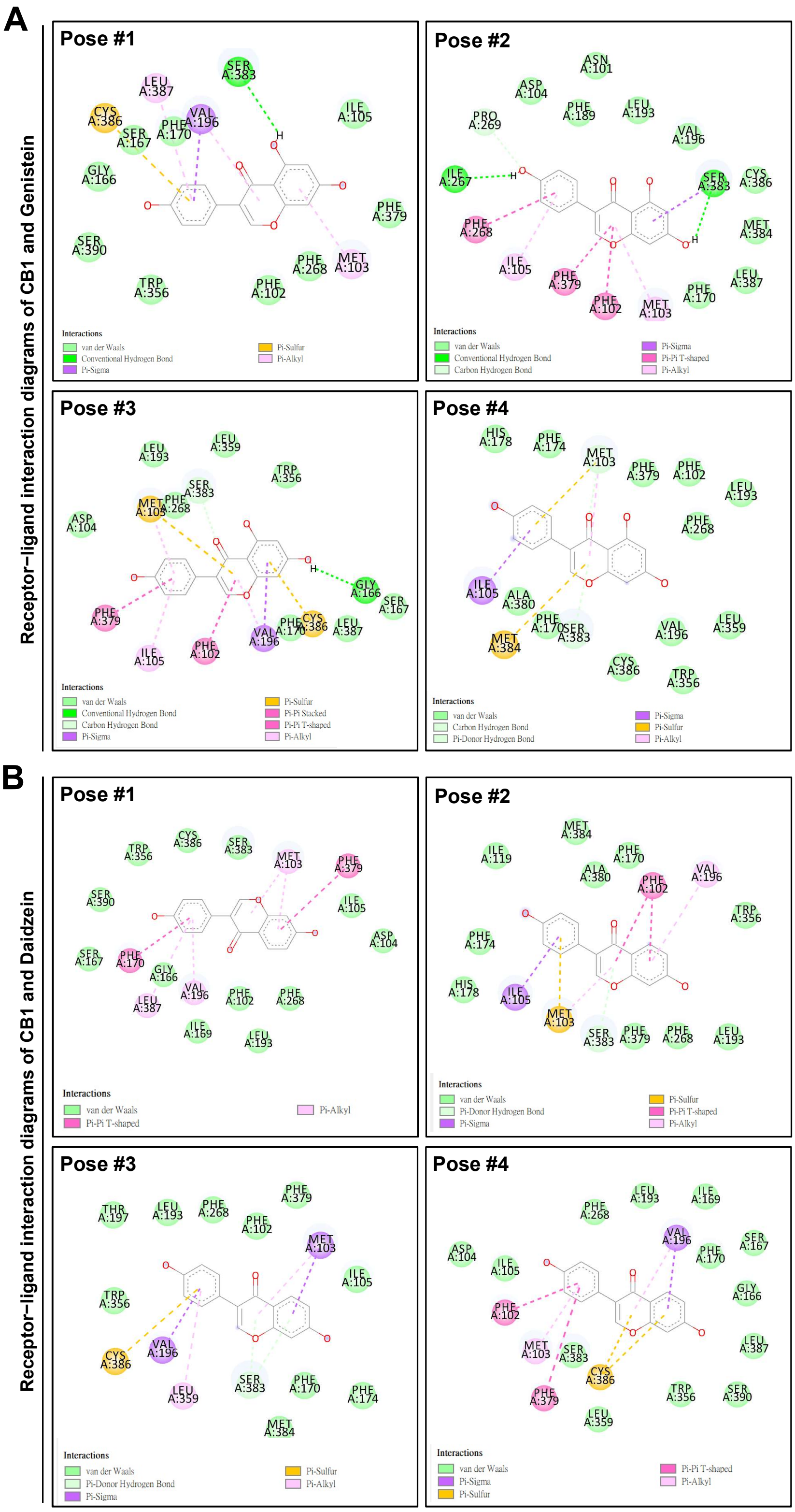

Figure S3. Conformations of genistein and daidzein docked within the binding site of the CB1 protein. (A) Binding interactions between genistein and amino acid residues in the CB1 protein. **(B)** Binding interactions between daidzein and amino acid residues in the CB1 protein.

Figure S4

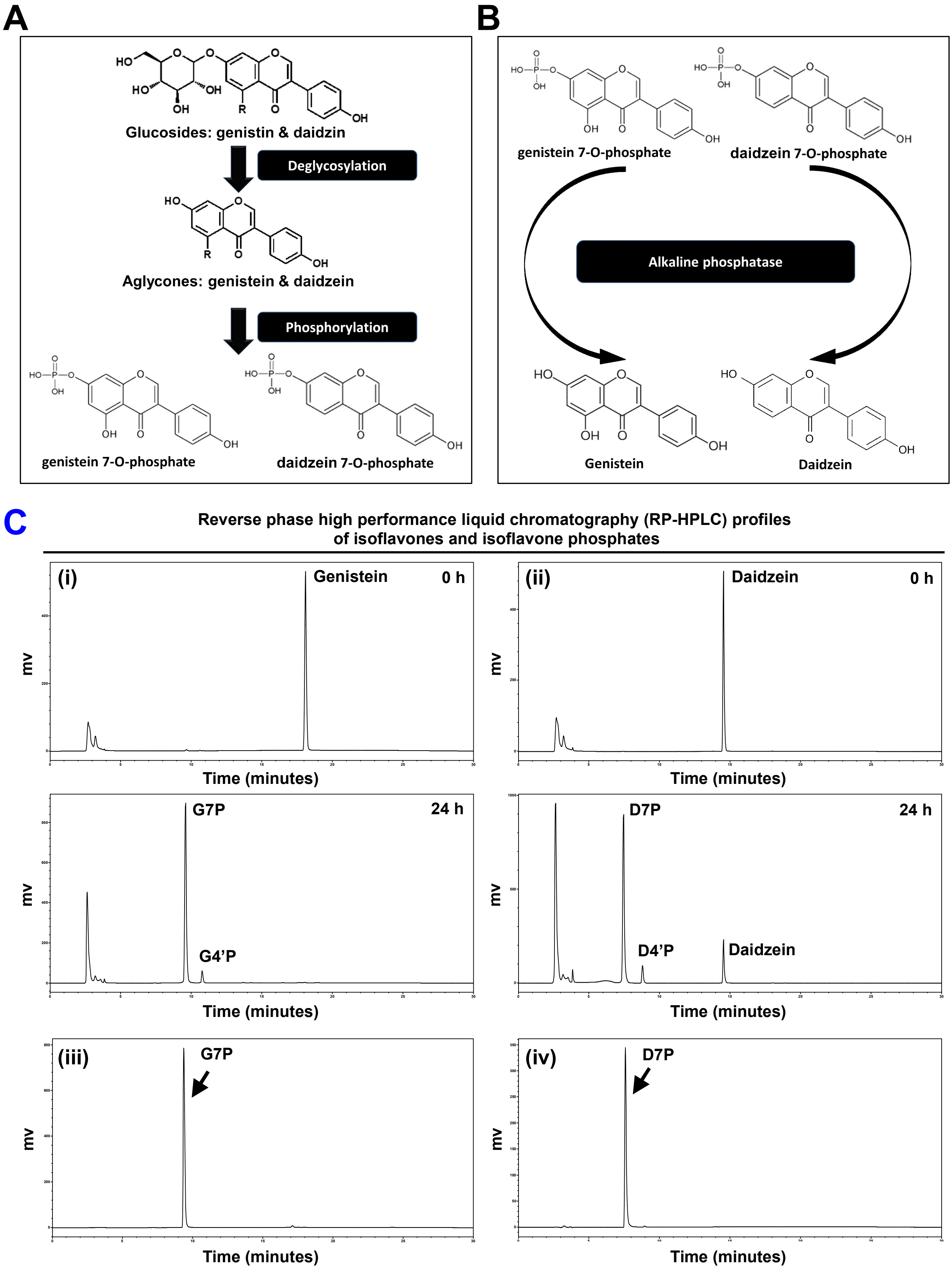
A
Glucosides: genistin & daidzin
Deglycosylation
Aglycones: genistein & daidzein
Phosphorylation
genistein 7-O-phosphate
daidzein 7-O-phosphate
B
genistein 7-O-phosphate
daidzein 7-O-phosphate
Alkaline phosphatase
Genistein
Daidzein
C
Reverse phase high performance liquid chromatography (RP-HPLC) profiles
of isoflavones and isoflavone phosphates
(i)
Genistein
0 h
(ii)
Daidzein
0 h
G7P
24 h
G4'P
D7P
24 h
D4'P
Daidzein
(iii)
G7P
(iv)
D7P
mv
Time (minutes)

Figure S4 (Continue)

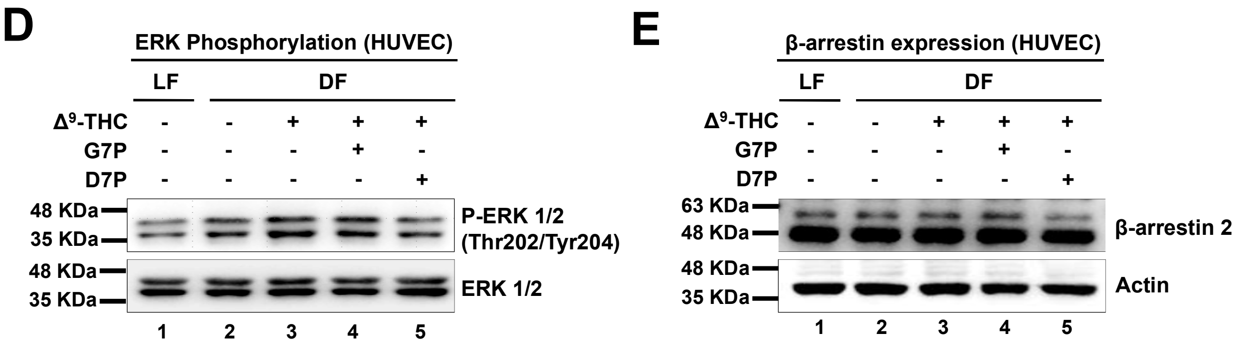

Figure S4. Genistein 7-O-phosphate and daidzein 7-O-phosphate are generated via a biotransformation-based strategy to improve water solubility and oral bioavailability. (A) Bioconversion of glucosidic isoflavones: genistin and daidzin are initially hydrolyzed to produce genistein and daidzein through deglucosylation, followed by subsequent phosphorylation to form genistein 7-O-phosphate (G7P) and daidzein 7-O-phosphate (D7P). **(B)** Phosphate esters of genistein and daidzein are dephosphorylated by alkaline phosphatase to generate their corresponding aglyconic isoflavones. **(C)** RP-HPLC chromatograms of the culture broth before and after the biotransformation of (i) genistein and (ii) daidzein by B. subtilis BCRC 80517 at 0 hour and 24 hours. Chromatograms of purified (iii) G7P powder (98.5% w/w purity) and (iv) D7P powder (95.2% w/w purity) are also shown. GEN, genistein; G7P, genistein 7-O-phosphate; G4'P, genistein 4'-O-phosphate; DAI, daidzein; D7P, daidzein 7-O-phosphate; D4'P, daidzein 4'-O-phosphate. The black arrowhead indicates the purified isoflavone phosphate products. **(D and E)** HUVECs were treated with 5 μM Δ^9-THC, 2 μM G7P, 2 μM D7P, or Δ^9-THC in combination with G7P or D7P for 24 hours under laminar flow (LF) or disturbed flow (DF) conditions. Total cell lysates were analyzed by Western blotting using the indicated antibodies.

Figure S5

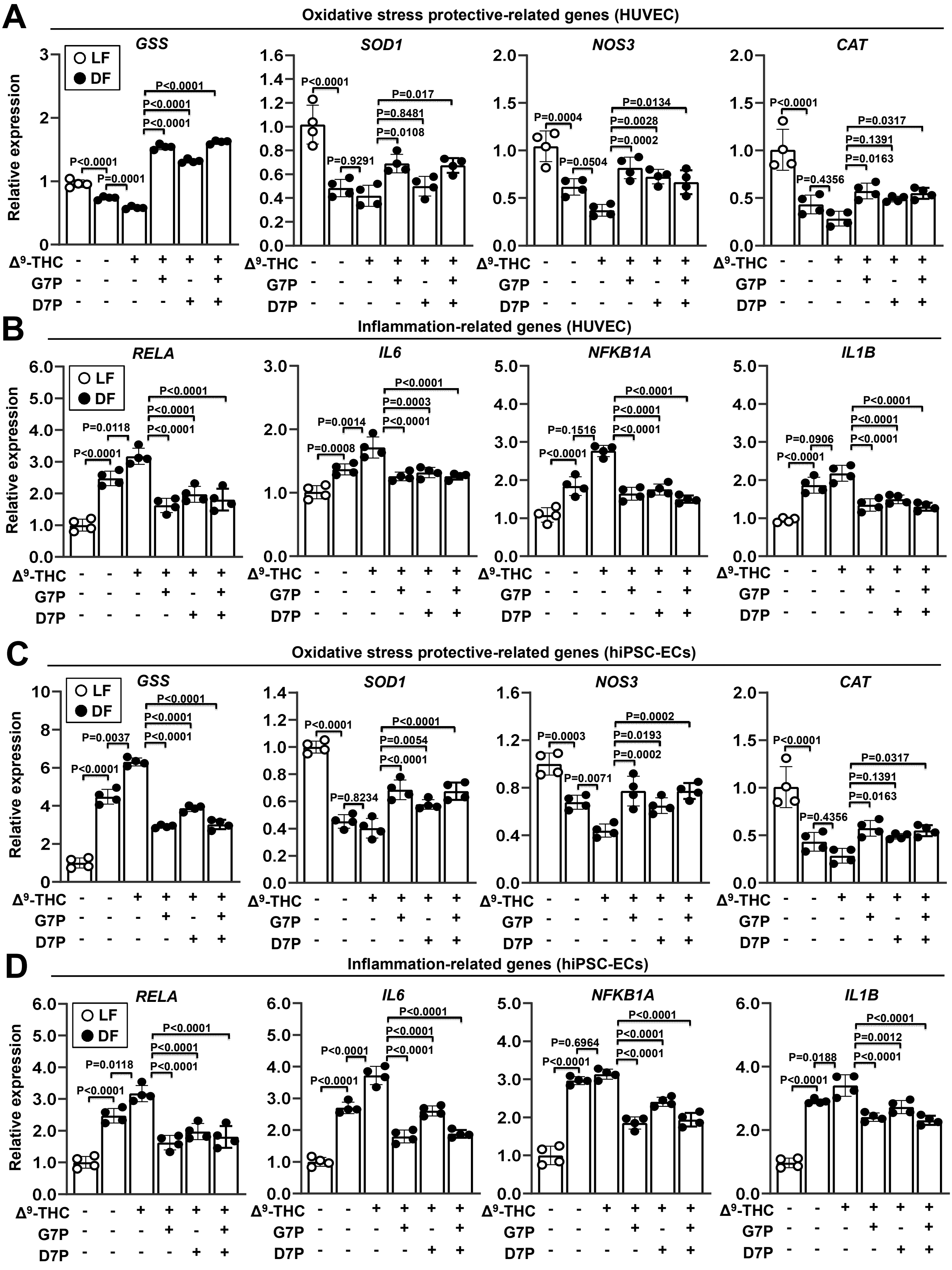

A
Oxidative stress protective-related genes (HUVEC)
GSS
SOD1
NOS3
CAT
LF
DF
Relative expression
Δ⁹-THC
G7P
D7P
B
Inflammation-related genes (HUVEC)
RELA
IL6
NFKB1A
IL1B
C
Oxidative stress protective-related genes (hiPSC-ECs)
D
Inflammation-related genes (hiPSC-ECs)

Figure S5 (Continue)

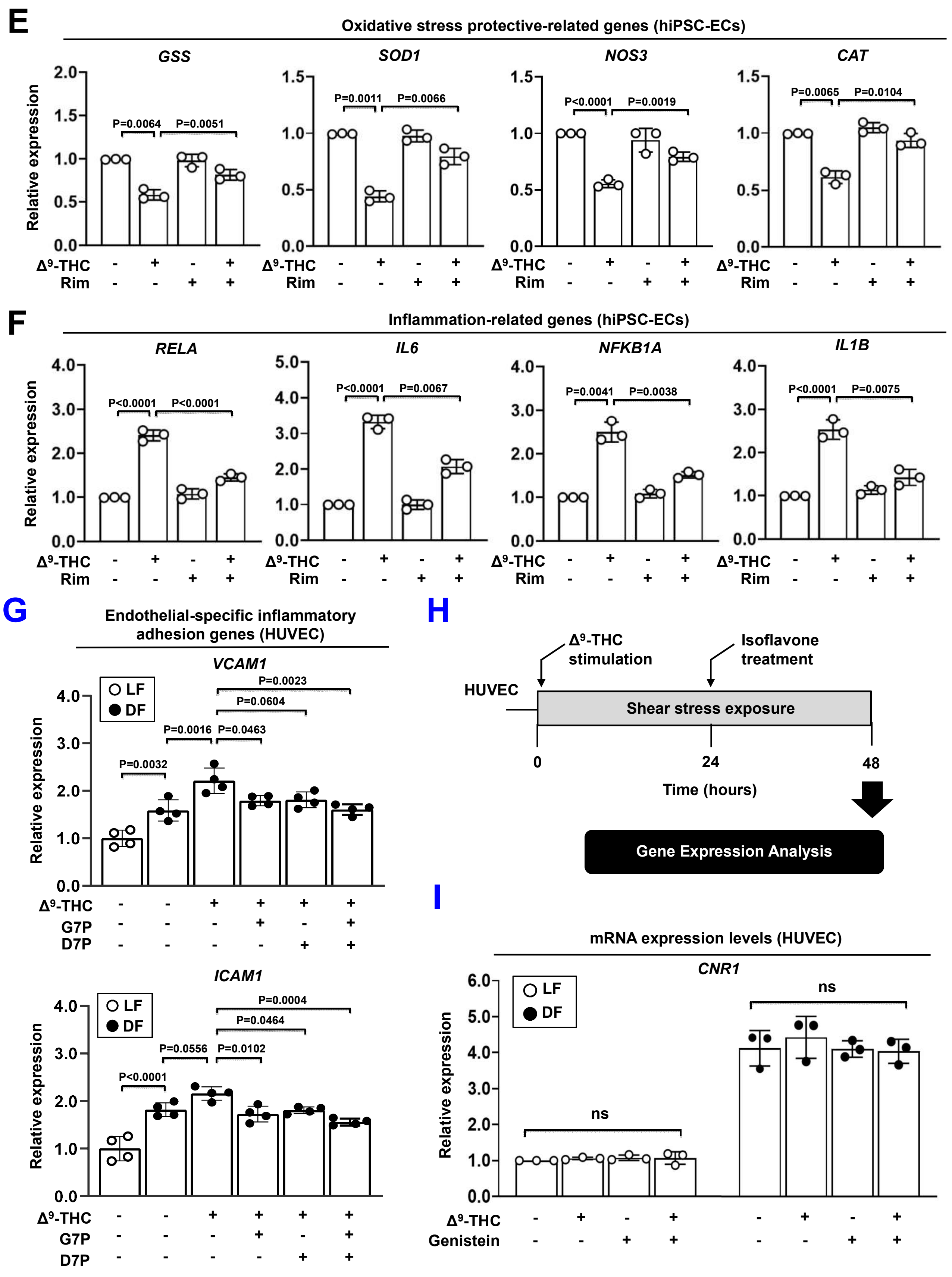

Figure S5. Effects of genistein 7-O-phosphate and daidzein 7-O-phosphate on oxidative stress and inflammation in endothelial cells. (A-D) HUVECs and hiPSC-ECs were treated with 5 μM Δ^9-THC, 2 μM G7P, 2 μM D7P, or a combination of G7P and D7P for 24 hours under laminar flow (LF) or disturbed flow (DF) conditions. mRNA expression levels were quantified by qPCR and normalized to GAPDH expression. **(A)** mRNA expression levels of oxidative stress-related protective genes in HUVEC were shown. **(B)** mRNA expression levels of inflammation-related genes in HUVEC were shown. **(C)** mRNA expression levels of oxidative stress-related protective genes in hiPSC-ECs were shown. **(D)** mRNA expression levels of inflammation-related genes in hiPSC-ECs were shown. **(E and F)** hiPSC-ECs were treated with 5 μM Δ^9-THC, 1 μM Rimonabant, or their combination for 24 hours under shear stress conditions. **(E)** mRNA expression levels of oxidative stress-related protective genes in hiPSC-ECs were shown. **(F)** mRNA expression levels of inflammation-related genes in hiPSC-ECs were shown. **(G)** Effects of G7P and D7P on endothelial-specific inflammatory adhesion genes in endothelial cells. HUVECs were treated with 5 μM Δ^9-THC, 2 μM G7P, 2 μM D7P, or a combination of G7P and D7P for 24 hours under shear stress conditions. mRNA expression levels were quantified by qPCR and normalized to GAPDH expression. **(H and I)** Effects of isoflavones on CNR1 expression in endothelial cells. HUVECs were cultured under shear stress for 24 hours, then treated with 5 μM Δ^9-THC, 10 μM genistein, or their combination for 24 hours. mRNA expression levels were quantified by qPCR and normalized to GAPDH. **(H)** Schematic overview of the experimental design. **(I)** CNR1 mRNA expression normalized to GAPDH. ns, no significance.

Figure S6

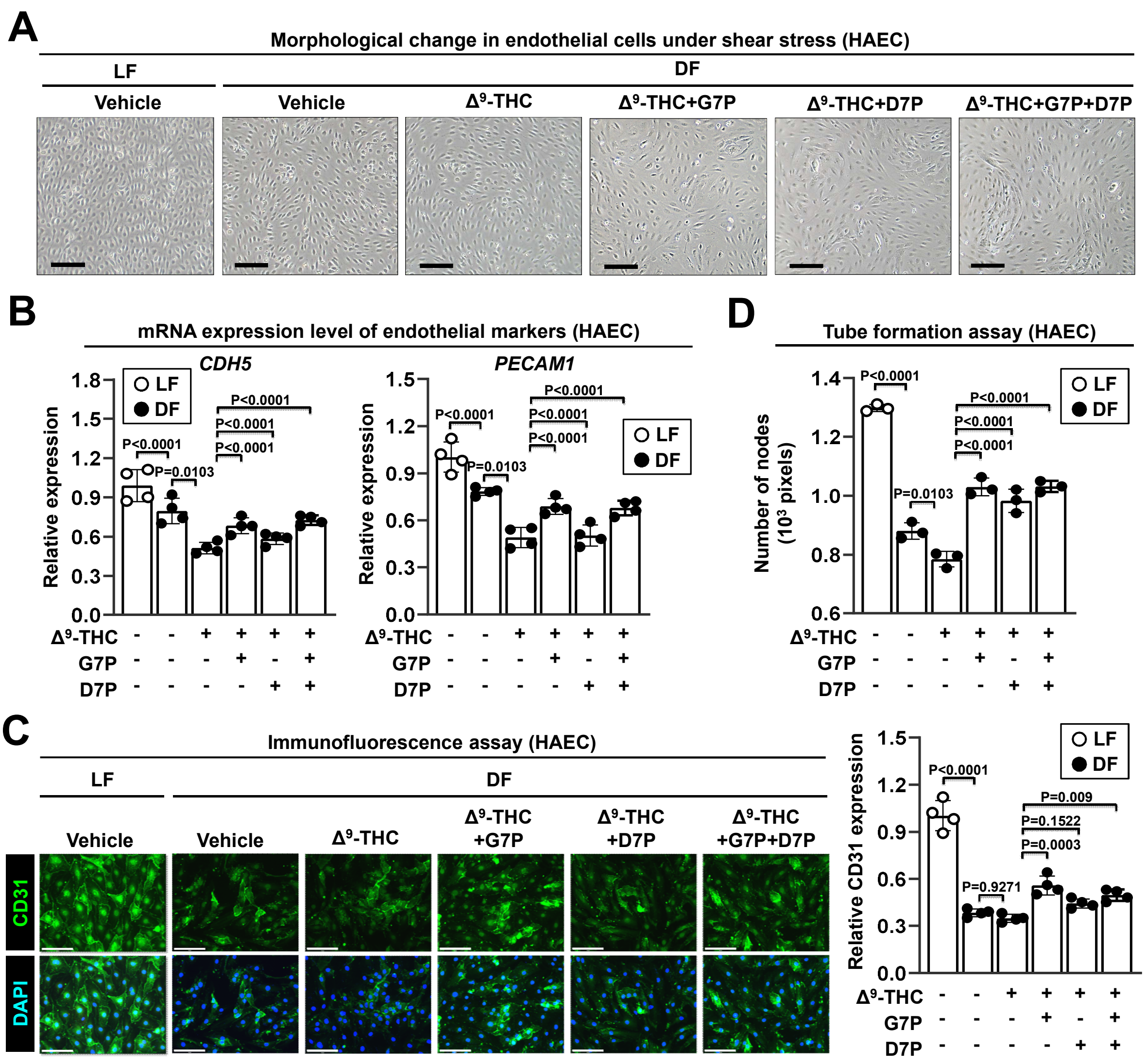

Figure S6. Effects of genistein 7-O-phosphate and daidzein 7-O-phosphate on endothelial functions in vascular endothelial cells. HAECs were treated with 5 μM Δ^9-THC, 2 μM G7P, 2 μM D7P, or a combination of G7P and D7P for 24 hours under laminar flow (LF) or disturbed flow (DF) conditions. **(A)** Morphology change of HAEC under shear stress conditions. Scale bar: 250 μm. **(B)** mRNA expression levels of endothelial markers were quantified by qPCR and normalized to GAPDH expression. **(C)** Immunofluorescence staining for CD31 (Alexa Fluor 488, green fluorescence) and nuclei (DAPI, blue fluorescence). Merged images are shown (left panel). Scale bar: 150 μm. Quantification of CD31 fluorescence intensity in HAEC was performed using ImageJ software (right panel). **(D)** Tube formation assays were performed on HAEC, and quantitative results are presented.

Figure S7

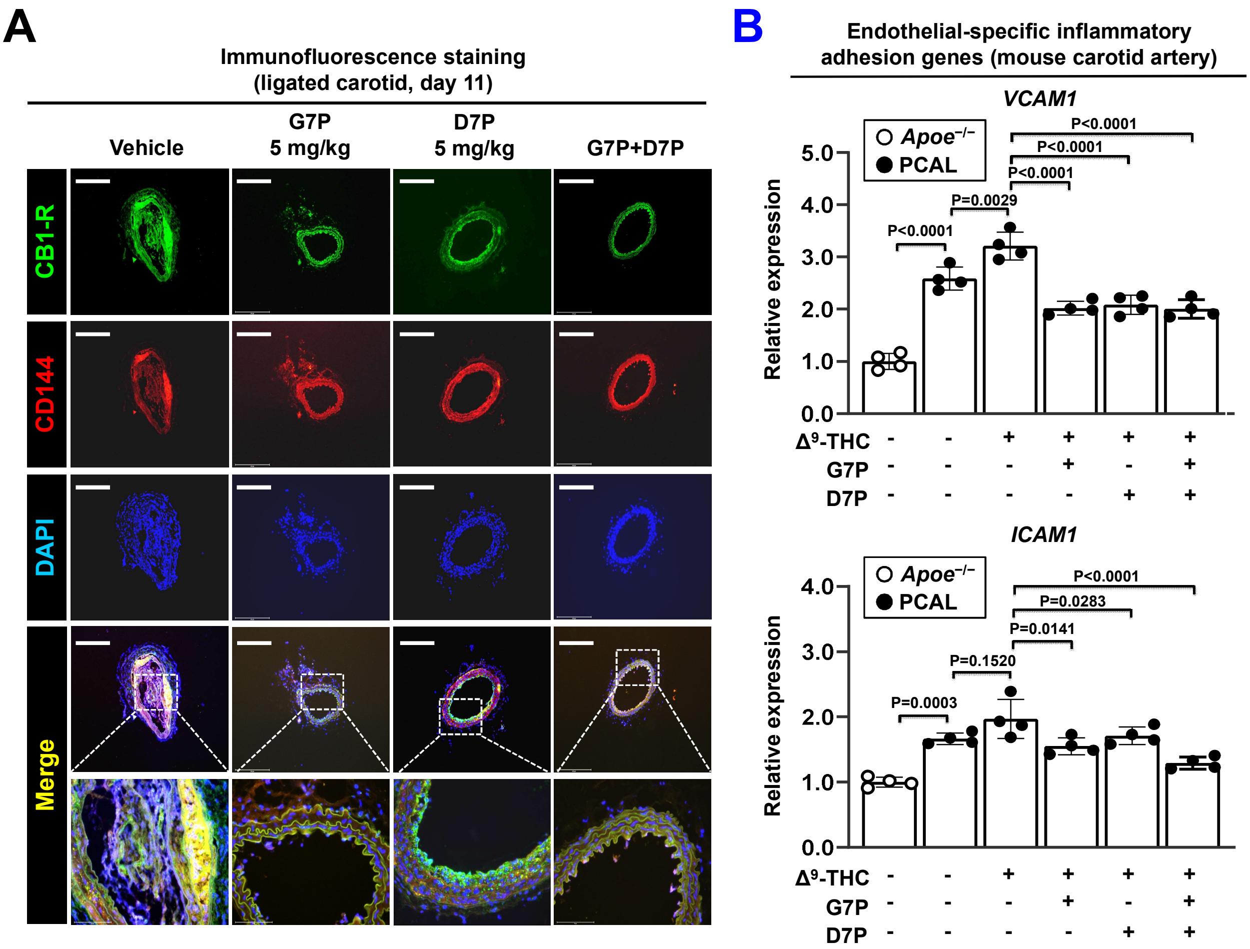

Figure S7. Effects of genistein 7-O-phosphate and daidzein 7-O-phosphate on inflammation in the endothelium of atherosclerosis mouse models. Partial carotid artery ligation (PCAL) was performed in Apoe$^{-/-}$ mice (8 weeks old). After PCAL, the Apoe$^{-/-}$ mice were divided into five groups: (1) vehicle control (n=5), (2) G7P (5 mg/kg; n=5), (3) D7P (5 mg/kg; n=5), and (4) G7P + D7P (n=5). All the groups received Δ^{9}-THC (1 mg/kg) via intraperitoneal injection twice daily for 10 days. Throughout the 12-day treatment period, the mice were maintained on a high-fat diet (HFD). At the end of the study, the mice were euthanized. **(A)** Carotid artery sections were immunostained with the indicated antibodies. Representative images for each group are shown, with high-magnification views of the regions outlined in white boxes. Scale bars: 275 μm. **(B)** Effects of G7P and D7P on endothelial inflammatory adhesion molecule expression in atherosclerotic mouse endothelium. mRNA expression levels of endothelial-specific inflammatory adhesion genes were quantified by qPCR and normalized to GAPDH expression.

Figure S8

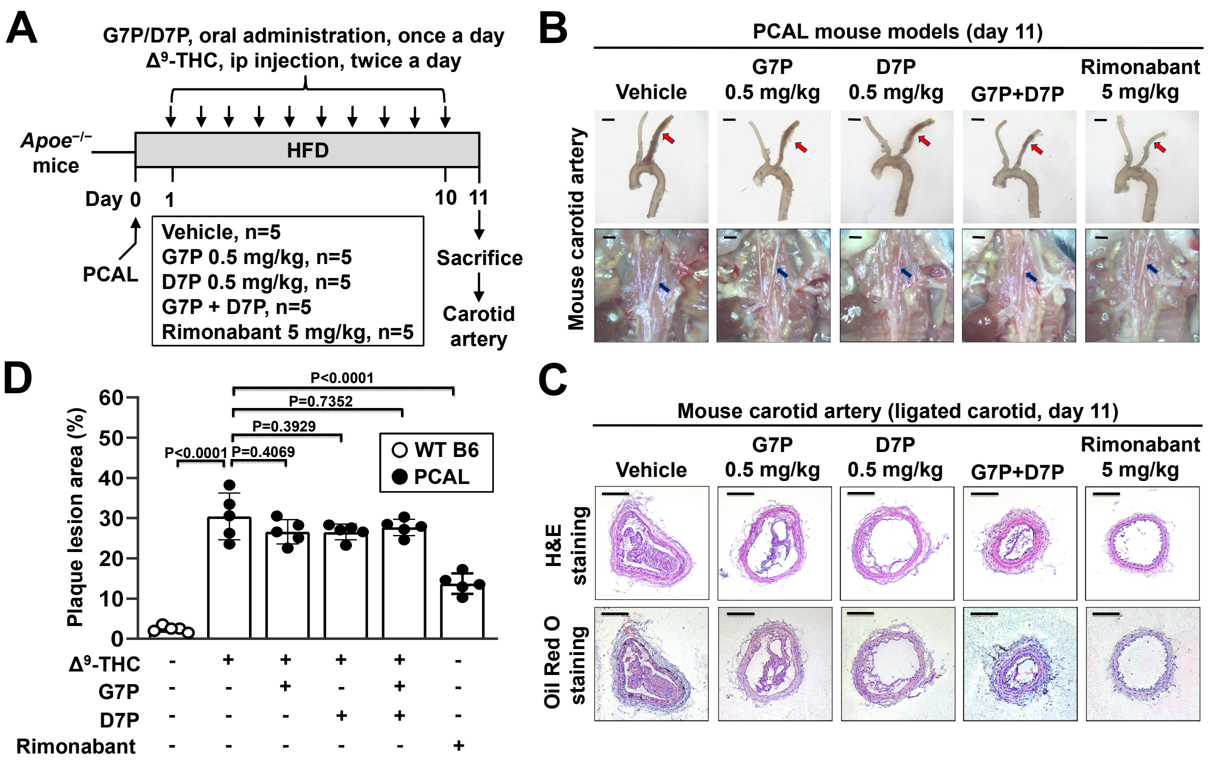

Figure S8. Effects of low-dose isoflavone monophosphates on atherosclerotic lesion progression in mice. Partial carotid artery ligation (PCAL) was performed in Apoe$^{-/-}$ mice (8 weeks old). After PCAL, the Apoe$^{-/-}$ mice were divided into five groups: (1) vehicle control (n=5), (2) G7P (n=5), (3) D7P (n=5), (4) G7P + D7P (n=5), and (5) Rimonabant (n=5). All the groups received Δ^{9}-THC (1 mg/kg) via intraperitoneal injection twice daily for 10 days. Throughout the 12-day treatment period, the mice were maintained on a high-fat diet (HFD). At the end of the study, the mice were euthanized. **(A)** Schematic overview of the experimental design. **(B)** Gross images of mouse carotid arteries are shown. The arrowhead indicates the ligated carotid artery. The scale bar represents 1 mm. **(C)** Carotid artery sections were counterstained with hematoxylin and eosin (H&E), and a representative slide is presented with scale bars at 275 μm (upper panel). Oil red O staining of atherosclerotic plaques in cross-sections of the mouse carotid artery (lower panel); scale bar, 275 μm. **(D)** Atherosclerotic plaques were quantified.

Figure S9

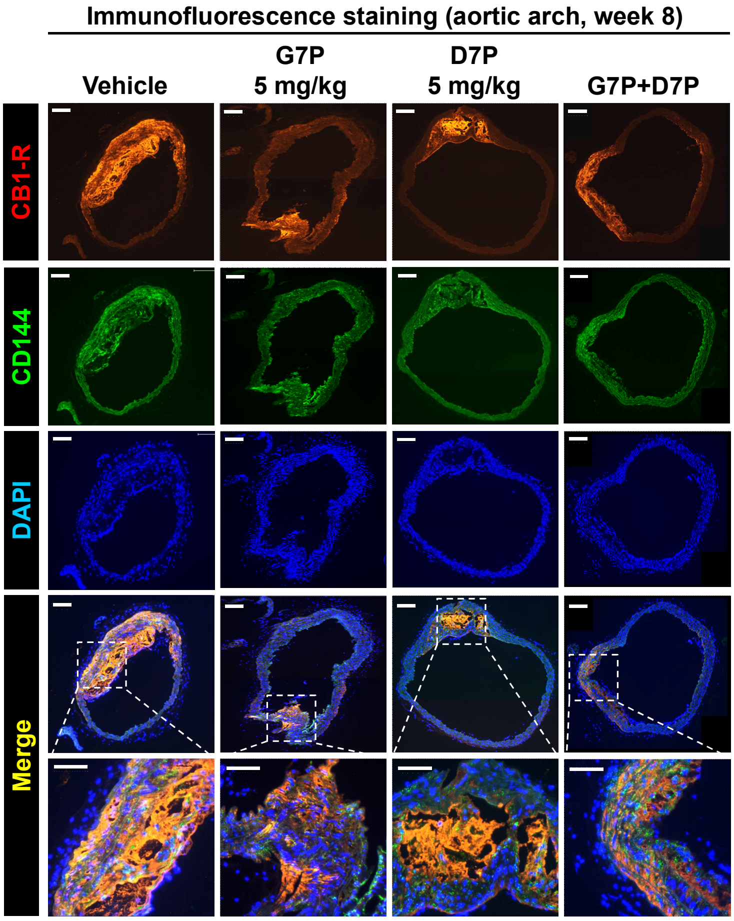

Figure S9. Effects of genistein 7-O-phosphate and daidzein 7-O-phosphate on CB1 expression in the endothelium of atherosclerosis mouse models. Apoe$^{-/-}$ mice (8 weeks old) were divided into four groups: (1) vehicle control (n=5), (2) G7P (5 mg/kg; n=5), (3) D7P (5 mg/kg; n=5), and (4) G7P + D7P (n=5). Mice were administered Δ^9-THC (1 mg/kg/day) subcutaneously via osmotic pumps, along with daily oral administration of G7P or D7P (5 mg/kg/day). All groups were fed a high-fat diet (HFD) throughout the 12-week treatment period. At the end of the study, mice were euthanized. Aortic arch sections were immunostained with the indicated antibodies. Representative images for each group are shown, with high-magnification views of the regions outlined in white boxes. Scale bars: 50 μm.

Figure S10

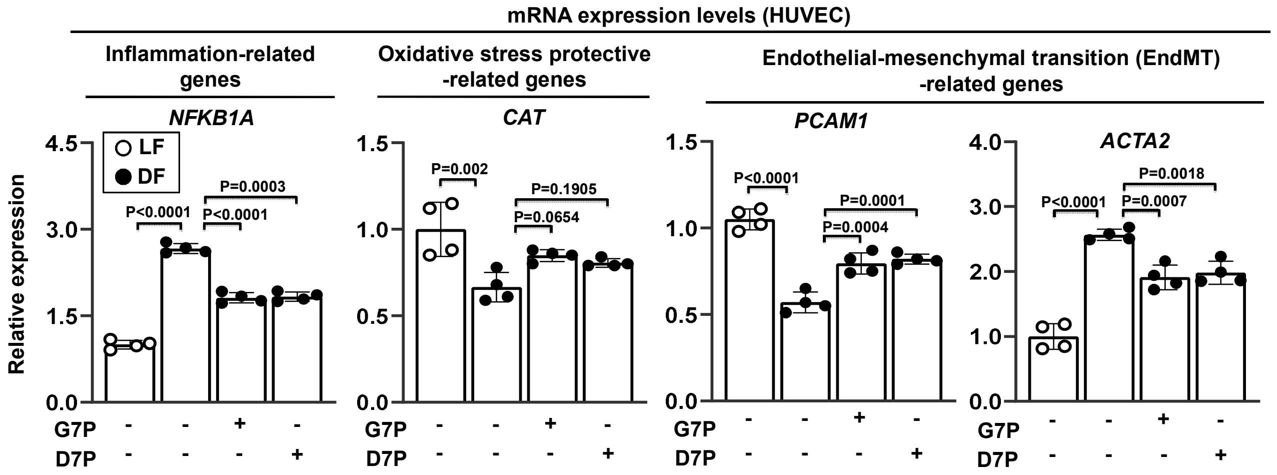

Figure S10. Effects of genistein 7-O-phosphate and daidzein 7-O-phosphate on inflammation, oxidative stress, and endothelial-to-mesenchymal transition in endothelial cells. HUVECs were treated with 2 μM G7P, 2 μM D7P, or a combination of G7P and D7P for 24 hours under shear stress. The expression of inflammation-related genes, oxidative stress protection-related genes, and endothelial-mesenchymal transition (EndMT)-related genes was measured by qPCR. mRNA expression was normalized to GAPDH expression.

Figure S11

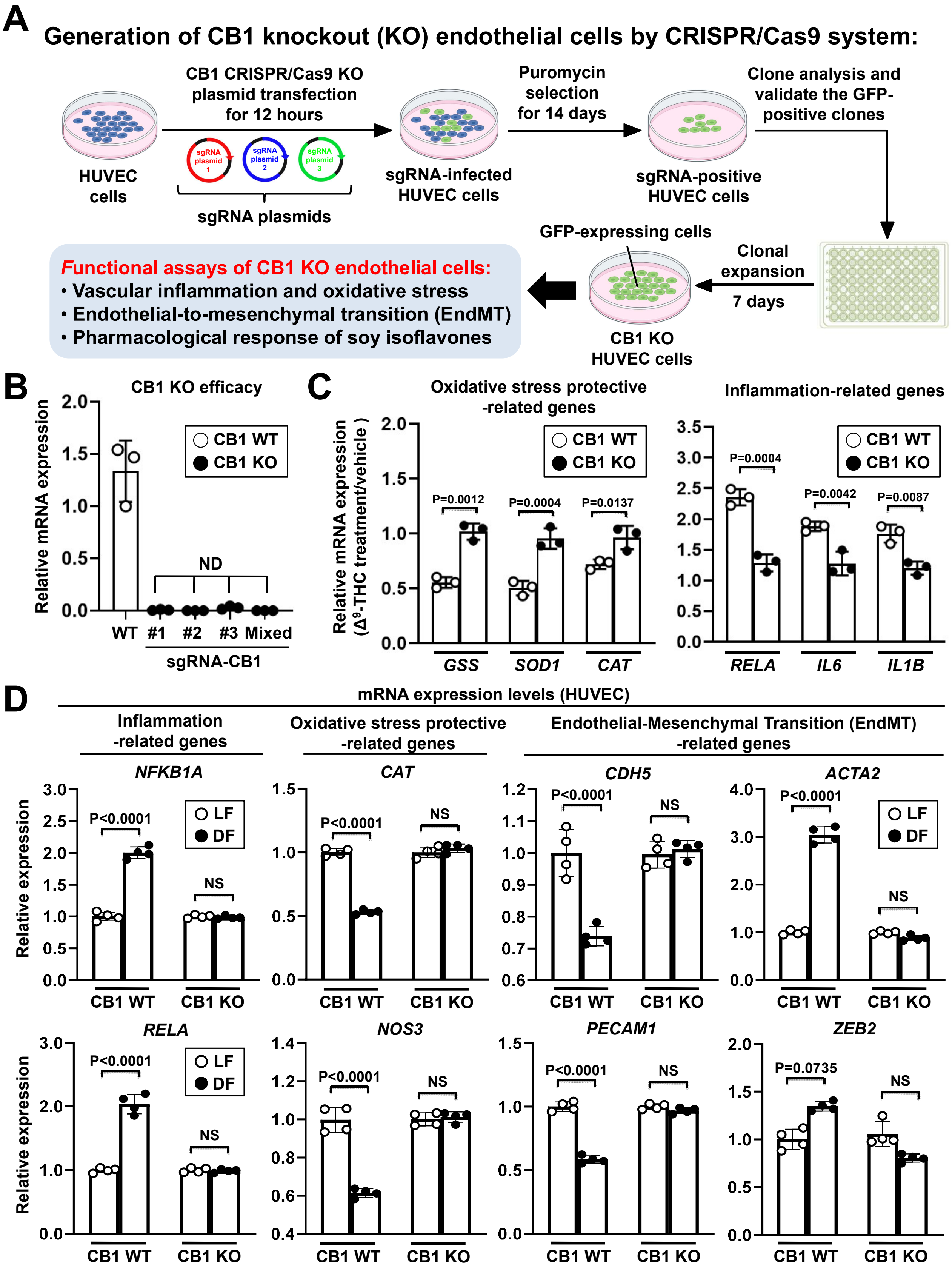
A
Generation of CB1 knockout (KO) endothelial cells by CRISPR/Cas9 system:
CB1 CRISPR/Cas9 KO plasmid transfection for 12 hours
HUVEC cells
sgRNA plasmids
sgRNA-infected HUVEC cells
Puromycin selection for 14 days
sgRNA-positive HUVEC cells
Clone analysis and validate the GFP-positive clones
GFP-expressing cells
Clonal expansion
7 days
CB1 KO HUVEC cells
Functional assays of CB1 KO endothelial cells:
• Vascular inflammation and oxidative stress
• Endothelial-to-mesenchymal transition (EndMT)
• Pharmacological response of soy isoflavones
B
CB1 KO efficacy
Relative mRNA expression
CB1 WT
CB1 KO
ND
WT #1 #2 #3 Mixed
sgRNA-CB1
C
Oxidative stress protective -related genes
Inflammation-related genes
Relative mRNA expression (Δ9-THC treatment/vehicle)
P=0.0012
P=0.0004
P=0.0137
P=0.0004
P=0.0042
P=0.0087
GSS
SOD1
CAT
RELA
IL6
IL1B
D
mRNA expression levels (HUVEC)
Inflammation -related genes
Oxidative stress protective -related genes
Endothelial-Mesenchymal Transition (EndMT) -related genes
NFKB1A
CAT
CDH5
ACTA2
RELA
NOS3
PECAM1
ZEB2
Relative expression
LF
DF
P<0.0001
P=0.0735
NS
CB1 WT
CB1 KO

Figure S11. Effects of knockout CB1 on endothelial functions in vascular endothelial cells. (A-C) Generation of CB1-deficient endothelial cells using the CRISPR-Cas9 system. **(A)** Schematic overview of the CRISPR/Cas9 system in HUVECs. CRISPR plasmids expressing Cas9 and CB1 sgRNAs were transfected into HUVECs for 12 hours. Cells were treated with puromycin (0.5 μg/ml) for 14 days. Single clones were picked according to GFP expression and expanded to generate monoclonal cell colonies. **(B)** The CNR1 mRNA levels in HUVECs treated with CB1 sgRNAs versus control were quantified by qPCR analysis. The mRNA expression was normalized to GAPDH. ND, not detected for 40 cycles by qPCR. **(C)** HUVECs were treated with 5 Mm Δ^9-THC for 48 hours. The mRNA expression of oxidative stress protective-related genes and inflammation-related genes in HUVECs treated with sgRNA versus control was quantified by qPCR analysis and normalized to GAPDH. **(D)** The HUVECs were transfected with either Control-shRNA or CB1-shRNA. CB1 WT and CB1 KO cells were cultured in an 6-well plate under laminar flow (LF) or disturbed flow (DF) conditions. The expression of inflammation-related genes, oxidative stress protection-related genes, and endothelial-mesenchymal transition (EndMT)-related genes was measured by qPCR. mRNA expression was normalized to GAPDH expression. NS, no significance.

Table S1. Primers for Real Time-Quantitative PCR.

Target gene	Primer set	Oligonucleotide sequence
ACTA2	Forward sequence	5’-CAGATCCAGACGCATGATGGCA-3’
	Reverse sequence	5’-GTAGACGACACCAGCAACAAGG-3’
CAT	Forward sequence	5’-GTGCGGAGATTCAACACTGCCA-3’
	Reverse sequence	5’-CGGCAATGTTCTCACACAGACG-3’
CDH5	Forward sequence	5’-GAAGCCTCTGATTGGCACAGTG-3’
	Reverse sequence	5’-TTTTGTGACTCGGAAGAACTGGC-3’
FN1	Forward sequence	5’-ACAACACCGAGGTGACTGAGAC-3’
	Reverse sequence	5’-GGACACAACGATGCTTCCTGAG-3’
GAPDH	Forward sequence	5’-GTCTCCTCTGACTTCAACAGCG-3’
	Reverse sequence	5’-ACCACCCTGTTGCTGTAGCCAA-3’
GSS	Forward sequence	5’-CCAAGACCGAAGGCTGTTTGTG-3’
	Reverse sequence	5’-TGTGACCTCTCCAGCAGTAGAC-3’
IL1B	Forward sequence	5’-CCACAGACCTTCCAGGAGAATG-3’
	Reverse sequence	5’-GTGCAGTTCAGTGATCGTACAGG-3’
IL6	Forward sequence	5’-AGACAGCCACTCACCTCTTCAG-3’
	Reverse sequence	5’-TTCTGCCAGTGCCTCTTTGCTG-3’
NFKB1A	Forward sequence	5’-TCCACTCCATCCTGAAGGCTAC-3’
	Reverse sequence	5’-CAAGGACACCAAAAGCTCCACG-3’
NOS3	Forward sequence	5’-GAAGGCGACAATCCTGTATGGC-3’
	Reverse sequence	5’-TGTTCGAGGGACACCACGTCAT-3’
PECAM1	Forward sequence	5’-AAGTGGAGTCCAGCCGCATATC-3’
	Reverse sequence	5’-ATGGAGCAGGACAGGTTCAGTC-3’
RELA	Forward sequence	5’-TGAACCGAAACTCTGGCAGCTG-3’

	Reverse sequence	5'-CATCAGCTTGCGAAAAGGAGCC-3'
SNAI1	Forward sequence	5'-TGCCCTCAAGATGCACATCCGA-3'
	Reverse sequence	5'-GGGACAGGAGAAGGGCTTCTC-3'
SNAI2	Forward sequence	5'-ATCTGCGGCAAGGCGTTTTCCA-3'
	Reverse sequence	5'-GAGCCCTCAGATTTGACCTGTC-3'
SOD1	Forward sequence	5'-CTCACTCTCAGGAGACCATTGC-3'
	Reverse sequence	5'-CCACAAGCCAAACGACTTCCAG-3'
TWIST1	Forward sequence	5'-GCCAGGTACATCGACTTCCTCT-3'
	Reverse sequence	5'-TCCATCCTCCAGACCGAGAAGG-3'
VIM	Forward sequence	5'-AGGCAAAGCAGGAGTCCACTGA-3'
	Reverse sequence	5'-ATCTGGCGTTCCAGGGACTCAT-3'
ZEB1	Forward sequence	5'-GGCATACACCTACTCAACTACGG-3'
	Reverse sequence	5'-TGGGCGGTGTAGAATCAGAGTC-3'
ZEB2	Forward sequence	5'-AATGCACAGAGTGTGGCAAGGC-3'
	Reverse sequence	5'-CTGCTGATGTGCGAACTGTAGG-3'

Table S2. List of antibodies used in this study.

Antibodies	Application	Dilution	Supplier name	Catalog number
Anti-α-smooth muscle actin Ab	IF	1:500	Abclonal	A17910
Anti-β-actin Ab	WB	1:5000	GeneTex	GTX109639
Anti-β arrestin 2 Ab	WB	1:1000	Abcam	ab314213
Anti-Cannabinoid receptor 1 Ab	IF	1:500	Abcam	Ab23703
Anti-ERK 1/2 mAb	WB	1:1000	Abclonal	A4782
Anti-KLF4 mAb	IP	1:500	Abclonal	A13673
Anti-NF-κB p65 Ab	IF	1:1000	Cell Signaling	8242
Anti-phospho-ERK 1/2 Ab	WB	1:1000	Cell Signaling	9101
Anti-Spi1 mAb	IP	1:500	Abcam	ab230336
Anti-VE-cadherin (CD144) Ab	IF	1:1000	Abclonal	A12416
Anti-ZNF610 Ab	IP	1:500	Invitrogen	PA5-68723

IF, immunofluorescence staining; WB, Western Blot; IP, immunoprecipitation.

Table S3. Radioligand binding assay of test compounds to the CB1 and CB2 receptors, related to Figure 3.

Compound	Organism	Target protein	IC_{50} value [b] (μM)	Ki value [b] (μM)
**Rimonabant** [a]	H. sapiens	CB1	0.0049	0.0045
**Daidzein**	H. sapiens	CB1	3.78	3.40
	H. sapiens	CB2	N.D. [c]	N.D. [c]

[a] Rimonabant is a well-known selective CB1 antagonist and is used as a positive control in radioligand binding assays. [b] IC_{50} and Ki values of test compounds by radioligand binding assays. [c] N.D., not determined due to low percent displacement at 50 μM in the radioligand binding assay.

Table S4. Predicted protein targets of genistein from the SwissTargetPrediction Database.

TARGETS [a]	COMMON NAME	TARGET CLASS
Thromboxane-A synthase	TBXAS1	Cytochrome P450
Monoamine oxidase A	MAOA	Oxidoreductase
Epidermal growth factor receptor	EGFR	Kinase
Estrogen receptor alpha	ESR1	Nuclear receptor
Maltase-glucoamylase	MGAM	Hydrolase
Serotonin 2a (5-HT2a) receptor	HTR2A	GPCRs [b]
Serotonin 2c (5-HT2c) receptor	HTR2C	GPCRs [b]
Adenosine A1 receptor	ADORA1	GPCRs [b]
Estrogen receptor beta	ESR2	Nuclear receptor
Adenosine A2a receptor	ADORA2A	GPCRs [b]
Estradiol 17-beta-dehydrogenase 1	HSD17B1	Enzyme
Estrogen-related receptor alpha	ESRRA	Nuclear receptor
Estrogen-related receptor beta	ESRRB	Nuclear receptor
ATP-binding cassette G2	ABCG2	Active transporter
Carbonic anhydrase VII	CA7	Lyase
Carbonic anhydrase XII	CA12	Lyase
Carbonic anhydrase IV	CA4	Lyase
Aldehyde dehydrogenase	ALDH2	Oxidoreductase
Cytochrome P450 19A1	CYP19A1	Cytochrome P450
Arachidonate 12-lipoxygenase	ALOX12	Enzyme

[a] Top ten predicted protein targets of genistein from the Swiss Target Prediction Database (http://www.swisstargetprediction.ch/). [b] Family A G protein-coupled receptors (GPCRs)

Table S5. Predicted protein targets of daidzein from the SwissTargetPrediction Database.

TARGETS [a]	COMMON NAME	TARGET CLASS
Aldehyde dehydrogenase	ALDH2	Oxidoreductase
Estrogen receptor alpha	ESR1	Nuclear receptor
Carbonic anhydrase VII	CA7	Lyase
Estrogen receptor beta	ESR2	Nuclear receptor
Carbonic anhydrase XII	CA12	Lyase
Carbonic anhydrase IV	CA4	Lyase
Thromboxane-A synthase	TBXAS1	Cytochrome P450
Monoamine oxidase A	MAOA	Oxidoreductase
Epidermal growth factor receptor	EGFR	Kinase
Maltase-glucoamylase	MGAM	Hydrolase
Serotonin 2a receptor	HTR2A	GPCRs [b]
Serotonin 2c receptor	HTR2C	GPCRs [b]
Adenosine A1 receptor	ADORA1	GPCRs [b]
Adenosine A2a receptor	ADORA2A	GPCRs [b]
Estradiol 17-beta-dehydrogenase 1	HSD17B1	Enzyme
Estrogen-related receptor alpha	ESRRA	Nuclear receptor
Estrogen-related receptor beta	ESRRB	Nuclear receptor
ATP-binding cassette G 2	ABCG2	Active transporter
Cytochrome P450 19A1	CYP19A1	Cytochrome P450
Arachidonate 12-lipoxygenase	ALOX12	Enzyme

[a] Top ten predicted protein targets of daidzein from the Swiss Target Prediction Database (http://www.swisstargetprediction.ch/). [b] Family A G protein-coupled receptors (GPCRs)

SUPPLEMENTARY MATERIALS AND METHODS

Reagents

Dimethyl sulfoxide (DMSO, D2650) and Formaldehyde solution 4% buffer (1.00496) were purchased from Sigma-Aldrich (Burlington, MA, USA).

HAEC, HUVEC, and THP-1 culture

Human Aortic Endothelial Cells (HAEC) were purchased from Lonza Bioscience (CC-2535, Basel, Switzerland). Human Umbilical Vein Endothelial Cells (HUVEC/TERT2, CRL-4053) and THP-1 cells (TIB-202) were obtained from the American Type Culture Collection (ATCC, Manassas, VA, USA). HAEC and HUVEC/TERT2 were cultured in Endothelial Growth Medium-2 (Lonza Bioscience, CC-3162), supplemented with 1% penicillin-streptomycin (Thermo Fisher Scientific, 15140122). THP-1 cells were cultured in RPMI 1640 medium (GIBCO, 11875085, Waltham, MA, USA) supplemented with 10% fetal bovine serum (FBS; Thermo Fisher Scientific, A5256701, Waltham, MA, USA), 1% penicillin-streptomycin, and 50 nM 2-mercaptoethanol (Thermo Fisher Scientific, 21985023). All cells were incubated at 37°C in a humidified incubator with 5% CO_2.

Differentiation of hiPSC-ECs

The hiPSC lines were generated by the Taiwan Human Disease iPSC Service

Consortium Resource Center (BCRC, SC81034, Hsinchu, Taiwan). These hiPSCs were maintained on GELTREX (Thermo Fisher Scientific, A1413201), and plated as single cells in StemFlex medium supplemented with ROCK inhibitor Y-27632 (SelleckChem, S1049, Houston, TX, USA). The hiPSC were cultured to 85% cell confluency and then treated for 2days with 6 mM CHIR99021 (SelleckChem, S2924) in RPMI medium plus B27 supplement without insulin (Thermo Fisher Scientific, A1895601). On day 2, the cells were treated for another 2 days with 2 mM CHIR99021 in RPMI plus B27 supplement without insulin. Subsequently, on day 4, the cells were treated with 10 ng/ml recombinant human fibroblast growth factor-2 (rhFGF-2) (Thermo Fisher Scientific, 13256-029) and 20 ng/ml recombinant human VEGF 165 protein (R&D Systems, 293-VE-050/CF, Minneapolis, MN, USA) for 8 days to expand the hiPSC-ECs. On day 12, the cells were harvested using Trypsin solution and filtered through a 30 μm cell strainer. The pellet was resuspended with 3 ml of cold MACS buffer per 10^7 cells, mixed with 20 μL anti-CD144 MicroBeads (Miltenyi Biotec, 130-097-857, Bergisch Gladbach, Germany), and incubated for 15 minutes at 4°C. The cells were then washed with cold MACS buffer, centrifuged at 300×g for 10 minutes, and applied to the MACS Separator column installed on the MACS MultiStand. The column was washed to flush out the labeled cells. The labeled hiPSC-ECs were cultured in gelatin-coated (Sigma Aldrich, G1393-100ML) 6-well

plates in EGM-2 medium.

Immunohistochemical (IHC) staining

IHC staining was performed by TnAlink Polymer Detection System (BIOTnA Biotech, TAHC04A, Kaohsiung City, Taiwan). Mouse tissues were fixed in 4% formaldehyde. The paraffin-embedded tissue slides were heated at 37°C incubator 1 hour and dewaxed in Sub-X xylene substitute (Leica Biosystems, 10015-094, Nussloch, Germany) 1 hour. The tissues were rehydrated in 100%, 95%, 90%, 80%, 70% alcohols and deionized water for 3 minutes each. The slides were then antigen retrieved with citric acid buffer at 95-100 °C for 20 minutes, followed by endogenous peroxidase block for 10 minutes and protein block for 90 minutes. The tissues incubated with primary antibody at 4°C overnight. The tissues were rinsed with PBS three times and incubated with TAlink Polymer for 1 hour. After three times PBS washed, the sections were covered with DAB solution and counterstained with hematoxylin. The slides were rinsed in running water for 15 minutes and were dehydrated by gradually increasing the concentration of alcohol and xylene, and finally coverslipped with mounting medium. The specimens were imaged via EVOS M7000 Imaging System (Thermo Fisher Scientific).

Monocyte adhesion assay

THP-1 cells (5×10^6) were labeled with CellTracker Fluorescent Probes (Thermo Fisher Scientific, C34551) for 45 minutes. The cells were then treated with the test compounds or cocultured with endothelial cells (HUVECs, HAECs, and hiPSC-ECs) that had been transduced with shZNF610, shSpi1, or KLF4 plasmids or an empty

vector. After 24 hours, the THP-1 cells were cocultured with the endothelial cells for 30 minutes at 37 °C. After incubation, the wells were washed three times with PBS to remove nonadherent cells. Images were acquired using the EVOS M7000 Imaging System. Spectrofluorometric quantification was performed on the number of adherent THP-1 cells using ImageJ software.

Tube formation assay

Endothelial cells (HUVECs, HAECs, and hiPSC-ECs) were seeded into 6-well plates and treated with test compounds under shear stress or transduced with the shZNF610, shSpi1, or KLF4 plasmids or an empty vector. The cells were maintained under shear stress conditions for 24 hours. The trypsinized cells were subsequently seeded into 96-well plates at a density of 1×10^4 cells per well on a Matrigel basement membrane matrix (Corning, Inc., 356234, Corning, NY, USA). Tube formation was assessed after 8 hours of incubation. Microscopic images were acquired, and the cumulative tube length was quantified using ImageJ software.

Gene transfection of endothelial cells

Endothelial cells were transfected with lentiviral vectors containing a puromycin resistance gene and polybrene (8 μg/ml). After transfection, the cells were selected with 2 μg/ml puromycin for 24 hours, followed by amplification with 1 μg/ml

puromycin for 7 days. The lentivirus expressing shRNA was generated with the pLKO vector, which contained scrambled shRNA (pLKO_TRC001, #ASN0000000004), pLKO_shZNF610 (#TRCN0000018048), and pLKO_shSpi1 (#TRCN0000417534). For the overexpression experiments, pMXs-hKLF4 (Addgene, Plasmid #17219, Watertown, MA, USA) and the empty pMXs vector (Cell Biolabs, Inc., RTV-010, San Diego, CA, USA) were transfected into endothelial cells using Lipofectamine 3000 (Thermo Fisher Scientific, L3000015). All the plasmids used for lentivirus production were provided by the National RNAi Core Facility, Academia Sinica (Taiwan).
